# People who use drugs show no increase in pre-existing T-cell cross-reactivity toward SARS-CoV-2 but develop a normal polyfunctional T-cell response after standard mRNA vaccination

**DOI:** 10.3389/fimmu.2023.1235210

**Published:** 2024-01-17

**Authors:** Murat Gainullin, Lorenzo Federico, Julie Røkke Osen, Viktoriia Chaban, Hassen Kared, Amin Alirezaylavasani, Fridtjof Lund-Johansen, Gull Wildendahl, Jon-Aksel Jacobsen, Hina Sarwar Anjum, Richard Stratford, Simen Tennøe, Brandon Malone, Trevor Clancy, John T. Vaage, Kathleen Henriksen, Linda Wüsthoff, Ludvig A. Munthe

**Affiliations:** ^1^ KG Jebsen Centre for B cell Malignancies, Institute of Clinical Medicine, University of Oslo, Oslo, Norway; ^2^ NEC OncoImmunity AS, Oslo, Norway; ^3^ Department of Immunology, Oslo University Hospital, Oslo, Norway; ^4^ ImmunoLingo Convergence Center, Institute of Clinical Medicine, University of Oslo, Oslo, Norway; ^5^ Institute of Clinical Medicine, University of Oslo, Oslo, Norway; ^6^ Agency for Social and Welfare Services, Oslo, Norway; ^7^ Student Health Services, University of Oslo, Oslo, Norway; ^8^ Unit for Clinical Research on Addictions, Oslo University Hospital, Oslo, Norway; ^9^ Norwegian Centre for Addiction Reasearch, Institute of Clinical Medicine, University of Oslo, Oslo, Norway

**Keywords:** people who use drugs, SARS-CoV-2 vaccination, T cell responses, T cell subsets, T cell functionality, antiviral immunity, opioids and immune responses

## Abstract

People who use drugs (PWUD) are at a high risk of contracting and developing severe coronavirus disease 2019 (COVID-19) and other infectious diseases due to their lifestyle, comorbidities, and the detrimental effects of opioids on cellular immunity. However, there is limited research on vaccine responses in PWUD, particularly regarding the role that T cells play in the immune response to severe acute respiratory syndrome coronavirus 2 (SARS-CoV-2). Here, we show that before vaccination, PWUD did not exhibit an increased frequency of preexisting cross-reactive T cells to SARS-CoV-2 and that, despite the inhibitory effects that opioids have on T-cell immunity, standard vaccination can elicit robust polyfunctional CD4^+^ and CD8^+^ T-cell responses that were similar to those found in controls. Our findings indicate that vaccination stimulates an effective immune response in PWUD and highlight targeted vaccination as an essential public health instrument for the control of COVID-19 and other infectious diseases in this group of high-risk patients.

## 1 Introduction

People who use drugs (PWUD) are at increased risk to be infected with severe acute respiratory syndrome coronavirus 2 (SARS-CoV-2) and present with severe coronavirus disease 2019 (COVID-19) due to possible immunosuppressive effects linked to their lifestyle (1, 2), higher rates of comorbidities (3, 4), and reduced average life span (5–10). Several studies expressed concerns regarding the potential of severe infection and greater mortality risks following SARS-CoV-2 infection in this group (11–21). Indeed, PWUD were at greater risk of being diagnosed with COVID-19, had a more severe course of the disease, and had greater COVID-19-related mortality (12, 22–25). Notably, the unfavorable immunological correlates of this high-risk group could be the result of direct and indirect effects of opioids on both innate and adaptive immune responses. An example of this is the suppressive effects that opioids have on antiviral T-cell responses, release of pro-inflammatory mediators, and antigen presentation (26–30).

Before vaccination, it became clear that a subset of the general population developed asymptomatic COVID-19 and that the responses in such settings could depend on T-cell cross-reactivity toward seasonal human coronaviruses (HCoVs) (31, 32). Thus, it was possible that the higher rate of community-acquired infections in PWUD may have increased T-cell immunity to seasonal HCoV and consequently augmented cross-reactive T-cell immunity toward SARS-CoV-2.

The importance of cellular immunity for a rapid and efficient resolution of COVID-19 has been emphasized in previous reports (33, 34). T cells are necessary for protection against severe COVID-19 before patients develop protective antibody responses (35) and for rapid viral clearance in patients who lack antibodies (36). Protective T-cell response in COVID-19 patients relies on appropriate T-cell phenotypes, and multiple correlates of protection have been extensively described in the literature (33, 34, 36, 37).

To date, T-cell immune response to vaccination has not been fully evaluated in PWUD: it is expected that the risk of co-morbidities and ongoing infections and the level of systemic inflammation and opioid use may contribute to reduced vaccine-specific T-cell activation in these individuals. We and others have shown that PWUD seroconvert after standard vaccination (38, 39), but whether PWUD can mount a vaccine-specific T-cell response remains to be determined. The clarification of this aspect of the immune response to SARS-CoV-2 vaccine is particularly important, as deranged T-cell immunity has been previously described in PWUD (26–30).

Here, we defined *a priori* T-cell immune response against a large set of SARS-CoV-2 peptides using Peripheral blood mononuclear cells (PBMC) collected in 2020 from non-exposed seronegative PWUD who were still antigenically naive to SARS-CoV-2. Then, we evaluated SARS-CoV-2 vaccine-generated polyfunctional T-cell response in PBMC collected the following year from another cohort of PWUD who received standard mRNA vaccination. Our data show no evidence of pre-existing SARS-CoV-2 cross-reactivity but indicate the existence of a robust polyfunctional T-cell response in PWUD after standard mRNA vaccination.

## 2 Materials and methods

### 2.1 Study design and participants

From 19 November 2020 to 9 February 2021, pre-vaccination blood was collected from a group of 19 people who use drugs (PWUD). All except one patient were HIV seronegative, and seen subjects received replacement therapy with methadone or buprenorphine. None of the patients presented detectable levels of anti-SARS-CoV-2 RBD or nucleocapsid IgG antibodies in the serum and had no history of COVID-19 infection. The post-vaccination cohort consisted of 25 PWUD donors. For this group, blood collection was performed in November 2021, 10–30 weeks after the participants had received their second dose of (mRNA) vaccines Moderna/mRNA-1273 or Pfizer/BioNTech BNT162b2. One of these donors received only one dose of mRNA vaccine but was diagnosed with SARS-CoV-2 46 days before blood sampling. One patient of this group had HIV and was on antiretroviral therapy, whereas eight subjects from this group belonged to the pre-vaccination cohort. Patients on medication-assisted rehabilitation were predominantly prescribed methadone or buprenorphine. See **Supplementary Tables S1**, **S2** for a summary of the clinical information and characteristics of all study participants. In addition, we tested 12 cryopreserved PBMC samples previously bio-banked from non-exposed seronegative healthy donors (HD) and five samples collected from COVID-19 convalescent donors. A cohort of 21 healthy subjects who also received two doses of mRNA vaccines was used as a control for post-vaccination studies.

### 2.2 Ethics approval

This study and all biobanking procedures carried on at Oslo University Hospital were approved by the Norwegian Regional Ethics Committee (approval nos. 166545 and 135924) and the Data Protection Officer at the Oslo municipality. Informed consent was obtained from all participants.

### 2.3 Samples and PBMC isolation

PBMC and serum samples were collected at a single time point for each patient (**Supplementary Table S2**).

PBMCs were isolated from the whole blood using either Cellular Preparation Tubes (CPT) tubes (BD vacutainer, # 362782) or Lymphoprep™ (Serumwerk Berburg, # 1858). CPT tubes were spun at 1,600*g* for 25 min at RT and plasma collected and stored at −20°C for future analysis. Lymphoprep™ isolation was performed according to the manufacturer’s instructions. Briefly, a 1:1 mix of blood and PBS (35 mL) was layered onto 10–15 mL Lymphoprep™ solution in a 50-mL Falcon tube. Tubes were then centrifuged for 25 min at 800*g* and PBMC collected, washed in PBS (Gibco, # 10010-015), and spun three more times (400*g*, 7 min, 4°C) before being counted and resuspended in Fetal bovine serum (FBS) (Gibco, # 10270-106) complemented with 10% Dimethyl sulfoxide (DMSO). After overnight pre-chilling at −80°C in Mr. Frosty (Nalgene™, # 5100-0001), cells were transferred to liquid nitrogen for long-term storage.

### 2.4 Serological analysis

IgG anti-RBD (BAU/mL) and IgG anti-nucleocapsid serum antibodies were quantified using a multiplexed bead-based assay as previously described (40).

### 2.5 Proliferation and IFNγ release in T-cell assays with single peptides

To test SARS-CoV-2-specific reactivity of T cells from pre-vaccinated donors, PBMC from each patient were distributed on a 96-well round bottom cell culture plate at a concentration of 3×10^5^ cells per well (~5×10^4^ and ~1×10^5^ CD8^+^ and CD4^+^ T cells, respectively) and stimulated with a pool of SARS-CoV-2 peptides (one peptide per well at 1.5 µg/mL: final volume = 200 µL per well; for the list of the 9–10-mer peptides used in these studies, see **Supplementary Methods**) (41, 42). At day 3, supernatants (SN) were collected for cytokine analysis (ELISA), after which methyl-^3^H thymidine (Hartmann Analytic GmbH, 0.02 mCi/mL) was added for cell proliferation assessment. After a 5- to 6-day incubation period, cells were harvested and counted using a PerkinElmer MicroBeta2 2450 Microplate Scintillation Counter. Proliferation data were calculated by subtracting the background signal (unstimulated cells). IFNγ ELISA was performed on supernatants using the following reagents: IFNγ monoclonal antibody (2G1, Invitrogen), Human IFNγ ELISA Standard Recombinant Protein (eBioscience), biotinylated IFNγ monoclonal antibody (B133.5, Invitrogen), horseradish peroxidase substrate TMB solution (Merck), and Streptavidin-rHRP (Southern Biotech). Plates were read with a Tecan multiplate reader. IFNγ levels were calculated by subtracting the background signal from unstimulated control wells or the average signal from the six wells with peptides that gave the lowest response.

### 2.6 SARS-CoV-2 peptide pools used in the study

Two collections of lyophilized 15-mer peptides with 11 amino acid overlaps were used in this study: one (PepTivator® SARS-CoV-2 Prot_S; Militenyi, # 130-126-700) covered the immunodominant regions (aa 304–338, 421–475, 492–519, 683–707, 741–770, 785–802, and 885–1,273), and the other (PepTivator® SARS-CoV-2 Prot_S Complete; Miltenyi, # 130-127-953) covered the entire length of the SARS-CoV-2 S glycoprotein (aa 5-1273; Protein QHD43416.1, GenBank MN908947.3). The combination of these two pools was named “Peptivator mix” and used to stimulate cells. In addition to the Peptivator mix, a collection of 101 9/10-mer spike and non-spike peptides identified by the NEC Immune Profiler (NIP) algorithm (43) was used to test CD8^+^ T-cell response. These epitopes were selected as top binders for the most prevalent HLA types in Norway, including HLA-A*01:01, HLA-A*02:01, HLA-A*03:01, HLA-A*23:0, HLA-A*29:02 HLA-B*07:02, HLA-B*08:01, HLA-B*15:01, HLA-B*15:02, HLA-B*40:01, HLA-B*44:02, HLA-C*03:03, HLA-C*04:01, HLA-C*07:01, and HLA-C*07:02. Among these peptides, 43 derived from the spike protein and 58 from non-spike regions (see **Supplementary Methods**). Additionally, a pool of 23 9/10-mer spike peptides (the NOI pool) validated in our laboratory (41, 42) was used to identify vaccine response. The list of peptides is shown in **Supplementary Methods**.

### 2.7 Flow cytometry and T-cell activation-induced marker assay

Quantification of T-cell activation was performed using the activation-induced marker assay (AIM) as described (41, 44–46). Briefly, stimulated PBMCs were washed in cold RPMI 1640 medium with GlutaMAX™ supplement and enriched in live cell using the magnetic column protocol according to manufacturer instructions (MACS MultiStand, # 130-042-303 with OctoMACS™ Separator). PBMCs (10 million cells per mL) were then distributed on a 96-well round bottom cell culture plate (200 μL/well) in TexMACS medium (Miltenyi, # 130-096-197) supplemented with 1 mmol/L Sodium Pyruvate (Gibco # 11360-039), 1 mmol/L MEM NEAA (Gibco # 11140-035), 50 nmol/L 1-thioglycerol (Sigma-Aldrich, # M1753), 12 μg/mL Gentamycin (VWR, # E737), and 20 U/mL IL-2 (R&D # AFL202). After 3 h incubation, cells were washed and treated (1 h) with 15-mer or 9/10-mer peptide pools (1.5 µg of peptide/mL in a final volume of 200 µL per well) in the presence of anti-CD28/CD49d co-stimulatory antibodies (BD # 347690) at a final concentration of 1:200. At this time, BV711 anti-human CD107a antibody (Clone H4A3; BioLegend, # 328640) was added for assessment of T-cell degranulation by flow. After an additional 18 h incubation with Brefeldin A/Monensin cocktail (GolgiStop 500X, Invitrogen # 00-4980-93), which improves assay sensitivity (47), cells were washed once in 1× PBS containing 5% FBS and 0.1% sodium azide, and then stained in the dark for 10 min with 0.5 μL of Fixable Near IR Live/Dead viability stain (Molecular Probes, # L34976) in a 10-μL final volume of cold PBS containing 5% FBS. Cells were then permeabilized at 4°C for 20 min in 100 μL BD Cytofix/Cytoperm solution (BD # 554714) and washed two times in 200 µL of 1× BD perm/wash solution (BD Biosciences Fixation and Permeabilization kit; # 554714). They were then stained in 20 µL of 1× BD perm/wash solution containing the following fluorochrome-conjugated antibodies: AF488 anti-human IL-2 (Clone MQ1-17H12; BioLegend, # 500314), PerCP-Cy5.5 anti-human CD8 (Clone RPA-T8; BioLegend, # 301032), PE anti-human CD137 (Clone 4B4-1; BioLegend, # 309804), PE-CF594 anti-human Granzyme B (Clone GB11; BD # 562462), PE-Cy5 anti-human (CD4 Clone RPA-T4; BD # 566925), PE-Cy7 anti-human TNF (Clone MAb11; Invitrogen, # 25-7349-82), AF647 anti-human IFNγ (BioLegend, #502516), BV510 anti-human CD40L (Biolegend, #310830), BV605 anti-human CD3 (Clone SK7; BD # 563219), and BV421 anti-human Perforin 1 (Clone dG9; BioLegend # 308122). Antibody concentrations were used according to manufacturer instructions. After a 30-min incubation in the dark, cell pellets were washed once in PBS, resuspended, and acquired on an Attune NxT Flow Cytometer (Thermo Fisher). Further replicate assays were performed similarly, but with a simplified staining panel (see **Supplementary Methods**).

### 2.8 T-cell response analysis

Responding T cells were identified using the “OR” Boolean gating (FlowJo’s Boolean analysis tools) that were found within regions double positive for the following marker pairs: CD137^+^ CD154^+^, CD154^+^ IFNγ ^+^, CD154^+^ IL-2^+^, CD154^+^ TNF^+^, TNF^+^ CD107a^+^, TNF^+^ GZMB^+^, TNF^+^ IFNγ^+^, or TNF^+^ Perforin^+^ (for gating strategy, see **Supplementary Figure S2**). Unstimulated background was subtracted to identify the frequency of specific activated T cells. Further in-depth functional profiling of SARS-CoV-2-specific T cells was performed using FlowJo’s Boolean analysis tools (AND, OR, and NOT), and data were presented with the Simplified Presentation of Incredible Complex Evaluations (SPICE) 6.1 software. Generated data were used to visualize antigen-specific T-cell polyfunctionality (48, 49), where activated cells expressed combinations of CD137, CD154, IFNγ, IL-2, and TNF (for the CD4+ Th cell subset) and CD107a, GZMB, IFNγ, Perforin, and TNF (for the CD8+ CTL cell subset). The frequency of all possible permutations above unstimulated background was calculated for responding specific T cells. For CTL, we performed the analysis on IFNγ+ or TNF+ cells (as subsets of CTL constitutively express GZMB and Perforin). Median frequencies of each population for each cohort were represented in a SPICE pie chart. A permutation test from the SPICE software was used to compare the distribution of cell populations for each pair of pie charts. Values of p < 0.05 were considered statistically significant. A detailed list of the possible permutations of polyfunctional populations can be found in **Supplementary Table S3**.

### 2.9 Statistical analysis

Statistical analyses were performed using GraphPad Prism V.9.3.1 (GraphPad software). Two-tailed Mann–Whitney U test, Spearman’s rank correlation coefficient, Principal Component Analysis (PCA) plots loading, and Pearson correlation matrix plots were used as indicated.

## 3 Results

### 3.1 T-cell responses to SARS-CoV-2 peptides in unexposed PWUD donors

**Figure 1:**
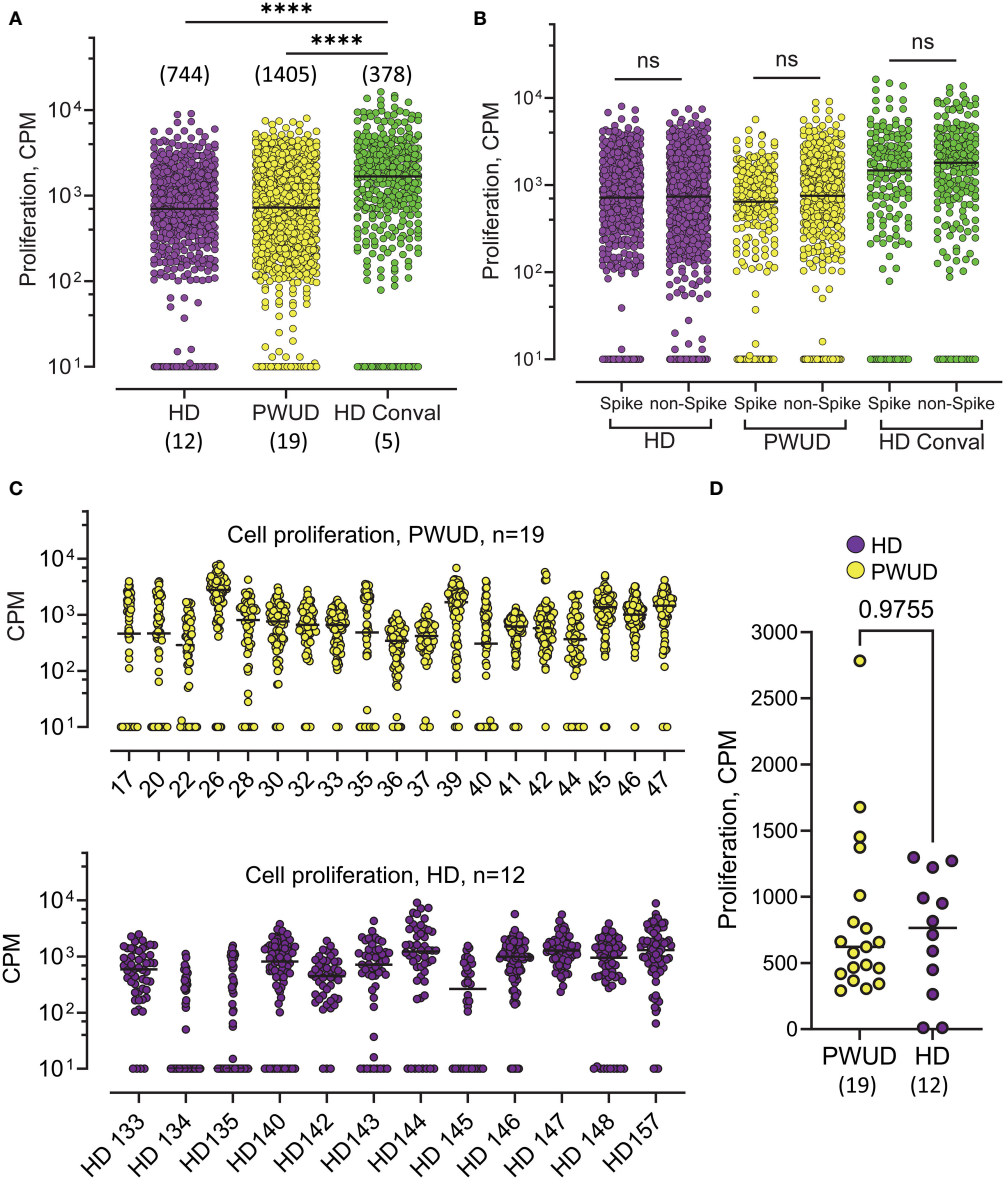
Proliferation of T cells in response to SARS-CoV-2 peptides in unvaccinated, non-infected individuals in the pre-vaccination era. **(A)** ^3^H Thymidine incorporation in PBMC isolated from PWUD (N=19), and HD (N = 12) donors. Samples were collected in 2020, when donors were no vaccinated, had no previous history of COVID-19, and were negative for IgG anti-RBD and IgG anti-nucleocapsid. Cells from each patient were stimulated with a pool of SARS-CoV-2 peptides (the complete list of the 101 peptides and HLA restriction is shown in **Supplementary Data**). Each datapoint represents the response to the stimulation with one peptide. The responses are pooled together for each cohort. Conval., Wuhan COVID-19 convalescent donors (N = 5); CPM, radioactivity counts per minute. Medians and significant differences are shown. Mann–Whitney U test, ****p < 0.0001. **(B)** ^3^H Thymidine incorporation data from **(A)** are shown with peptides regrouped according to protein of origin (Spike vs. non-Spike). Spike peptides (from Spike protein; N = 43) and non-Spike peptides (from ORF1ab, ORF3A, and Envelope proteins; n = 57). Not significant; Mann–Whitney U test, p > 0.05. **(C)** Response breakdown by donor. Response to each peptide from **(A)** are shown for each donor of the PWUD (top) and HD (bottom) cohort. **(D)** Median response to peptide stimulation is shown for each donor. Not significant; Mann–Whitney U test, p > 0.05. See also **Supplementary Figure S1** for the corresponding IFNγ response.

In 2020, public health concerns during the height of the COVID-19 pandemic led us to collect blood samples to investigate humoral responses in PWUD. We found that only 4 of 99 PWUD developed an IgG anti-RBD response in the months before vaccines were available (not shown), a rate that was similar to what we have found in the general population (39). Of the 36 PWUD donors who participated in the current study, 28 used illegal or prescribed opioids, 19 were in the pre-vaccination group, and 25 were in the post-vaccination group. Both pre- and post-vaccination samples were available for eight donors, one of whom received only one dose of vaccine but was diagnosed with SARS-CoV-2 46 days before blood was sampled (see *Materials and Methods*, **Supplementary Tables S1, S2**). We first tested T-cell responses in unexposed PWUD and healthy donors (HD) who were not vaccinated and were seronegative for anti-RBD and anti-nucleocapsid antibodies. In these groups, a response would likely be the result of cross-reactivity of T cells generated from prior exposure to endemic seasonal HCoV. We tested T-cell response to single peptides derived from spike (N = 43) or non-spike proteins (ORF1ab, ORF3A, Envelope; N = 57) in three sub-cohorts: PWUD (N = 19), HD (N = 12), and, as positive controls, COVID-19 Wuhan convalescent patients (N = 5; see *Materials and methods* and **Supplementary Methods** for peptide list). Overall, we found that proliferation (**Figure 1**) and IFNγ secretion (**Supplementary Figure S1A**) were both significantly increased in convalescent donors and that although the proliferative response was similar between PWUD and HD donors, PWUD showed a slight decrease in IFNγ secretion (**Figure 1**, **Supplementary Figure S1A**). In all the cohorts, the level of the proliferative response and IFNγ secretion was comparable notwithstanding the type of stimulus (spike vs. non-spike peptides; **Figure 1B**, **Supplementary Figure 1B**). As expected, donors in the PWUD and HD cohorts presented with variable degrees of proliferative and secretory response (**Figure 1C**, **Supplementary Figure 1C**), but there was no significant difference between groups (**Figure 1D**; 621 vs. 746.5 CPM median values, p = 0.98 and **Supplementary Figure S1D**; 13.2 vs. 22.8 pg/mL median values, p = 0.42) indicating that PWUD do not possess T-cell cross-reactivity generated from potential prior exposures to endemic seasonal HCoV.

### 3.2 Vaccine-generated CD4 Th cell response in PWUD

**Figure 2:**
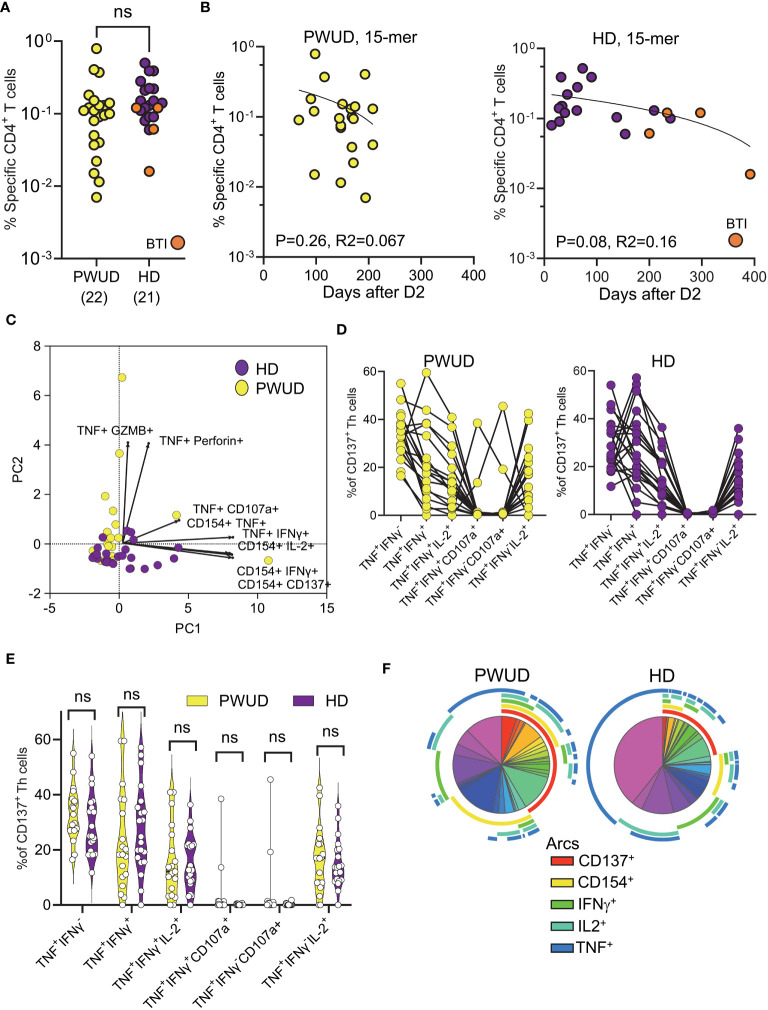
Spike-specific CD4 Th cell responses in vaccinated donors. Response of CD4 Th cells (% response – background frequency) quantified by flow cytometry after 18 h stimulation with the Peptivator mix. **(A)** Frequency of responder cells gated by Boolean analysis (OR) within the double positive regions (see **Supplementary Figure S2**) is shown for each patient (PWUD vs. HD); Mann–Whitney U test; p > 0.05, not significant (ns). BTI, breakthrough infection. **(B)** Data from **(A)** are plotted in function of time after vaccination for both PWUD (left) and HD (right) donors. The correlative trend was not significant. **(C)** PCA analysis and loading plot dimensions for PWUD and HD donors. **(D)** T-cell polyfunctionality in PWUD (left) and HD (right) donors quantified as % of CD137^+^ CD4 Th cells expressing different marker combinations. **(E)** Violin plots of data from **(D)** are shown; Mann–Whitney U test, p > 0.05 for all comparisons; ns. **(F)** Boolean analysis of the polyfunctional responses of CD4 Th cells visualized for PWUD and HD donors using SPICE 6. Pie charts represent the median frequency of T-cell-expressing combinations of the indicated markers after 18 h overnight stimulation with the Peptivator mix: the arcs indicate the proportion of cells that express CD137 (red), CD154 (yellow), IFNγ (green), IL-2 (light blue), and TNF (blue). The results for PWUD and HD donors are shown on the right and on the left, respectively. A detailed list of the permutations represented by the pie segments can be found in **Supplementary Table S3**. BTI, breakthrough infection.

In 2021, as vaccine rollout continued, we managed to secure follow-up blood samples from 25 PWUD donors (**Supplementary Tables S1, S2**). Due to difficulties in recalling donors for sampling, blood was drawn between 67 and 208 days after the second dose (D2) and not after the optimal 30-day interval. A similar approach was thus employed to gather blood samples from healthy donors in 2022, but due to the surge of Omicron, the likelihood of breakthrough infection (BTI) and hybrid immunity in this control cohort was significantly increased. Indeed, four of these controls experienced breakthrough infection before collection. To assess vaccine-induced T-cell activation, we challenged PBMC with commercially available 15-mer spike peptide megapool mixes (Peptivator mix; see *Materials and methods*) and measured by flow cytometry the increase in the frequency of T-cell populations that were double positive for different combinations of selected activation-induced markers (AIMs). Gating strategy and AIMs used in the study are reported in **Supplementary Figure S2**. We assessed the magnitude of spike-specific responses by determining the frequencies of cells that were double positive over background for one or more of the following marker pairs by “OR” Boolean gating: CD137^+^ CD154^+^, CD154^+^IFNγ^+^, CD154^+^ IL-2^+^, CD154^+^ TNF^+^, TNF^+^ CD107a^+^, TNF^+^ GZMB^+^, TNF^+^ IFNγ^+^, or TNF^+^Perforin^+^ (see **Supplementary Figure S2** for gates). Frequencies of responding spike-specific cells showed that the level of response in PWUD and HD donors was not significantly different (**Figure 2A**). As sampling time was variable after vaccination in PWUD, we also visualized T-cell responses vs. time post-vaccination, but found no significant correlations in PWUD or HD (**Figure 2B**). HD controls were chosen to resemble PWUD in terms of time since vaccination, but after sampling, four were found to have had breakthrough infection (BTI) with Omicron VOC (as they were positive for IgG anti-Nucleocapsid) (**Figures 2A, B**). Furthermore, when marker combinations were taken separately, we observed that the cytotoxic response in PWUD (frequency increase in TNF^+^ GZMB^+^ events) was higher in PWUD than in HD, but that for other AIM combinations, such as those that included the expression of CD154 and TNF/IFNγ co-expression, the response was higher in HD. Moreover, these results did not appear to be influenced by the four donors with BTI in the control group (**Supplementary Figure S3**). Representation by Principal Component Analysis (PCA) showed substantial overlap between cohorts and confirmed that, with the exception of a few PWUD patients who showed enhanced cytotoxic response (increased upregulation in perforin, granzyme B, and CD107a), CD4 Th cell responses in PWUD were similar to the response of the healthy subjects (**Figure 2C**). Further analysis revealed that the pattern of Th cell polyfunctionality in PWUD was not significantly different from the one observed in HD (**Figure 2D**). More specifically, we found that among all vaccine-specific, activated (CD137^+^) Th cells, the fraction of events that were double or triple positive for TNF, IFNγ, and/or IL-2^+^ were not significantly different between PWUD and HD (**Figure 2E**; 33% 18%, 12%, and 17% vs. 25%, 25%, 13%, and 14% median frequency values for TNF^+^ IFNγ^+^, TNF^+^ IFNγ^+^ IL-2^+^, and TNF^+^ IFNγ^−^ IL-2^+^ populations in PWUD and HD, respectively; Mann–Whitney U test, p > 0.05). A similar analysis of activated CD154^+^ Th cells produced similar results (data not shown). These data were further confirmed by Pearson correlation analysis, which showed that the correlation patterns among different expression markers measured in the two cohorts were similar (**Supplementary Figure S4A**). A comprehensive representation of the polyfunctional response is shown for both cohorts in **Figure 2F**, where we employed the SPICE tool to visualize and compare frequencies of single, double, triple, and quadruple positive cells within the activated CD4 Th cell compartment (see *Materials and methods*). This analysis confirmed the polyfunctional nature of the response in PWUD and showed that although the frequency of some vaccine-specific T-cell populations was lower in PWUD, the pattern of CD4 Th cell polyfunctionality was not significantly different from the one observed in healthy controls.

### 3.3 Vaccine-generated CD8 CTL T-cell response in PWUD

**Figure 3:**
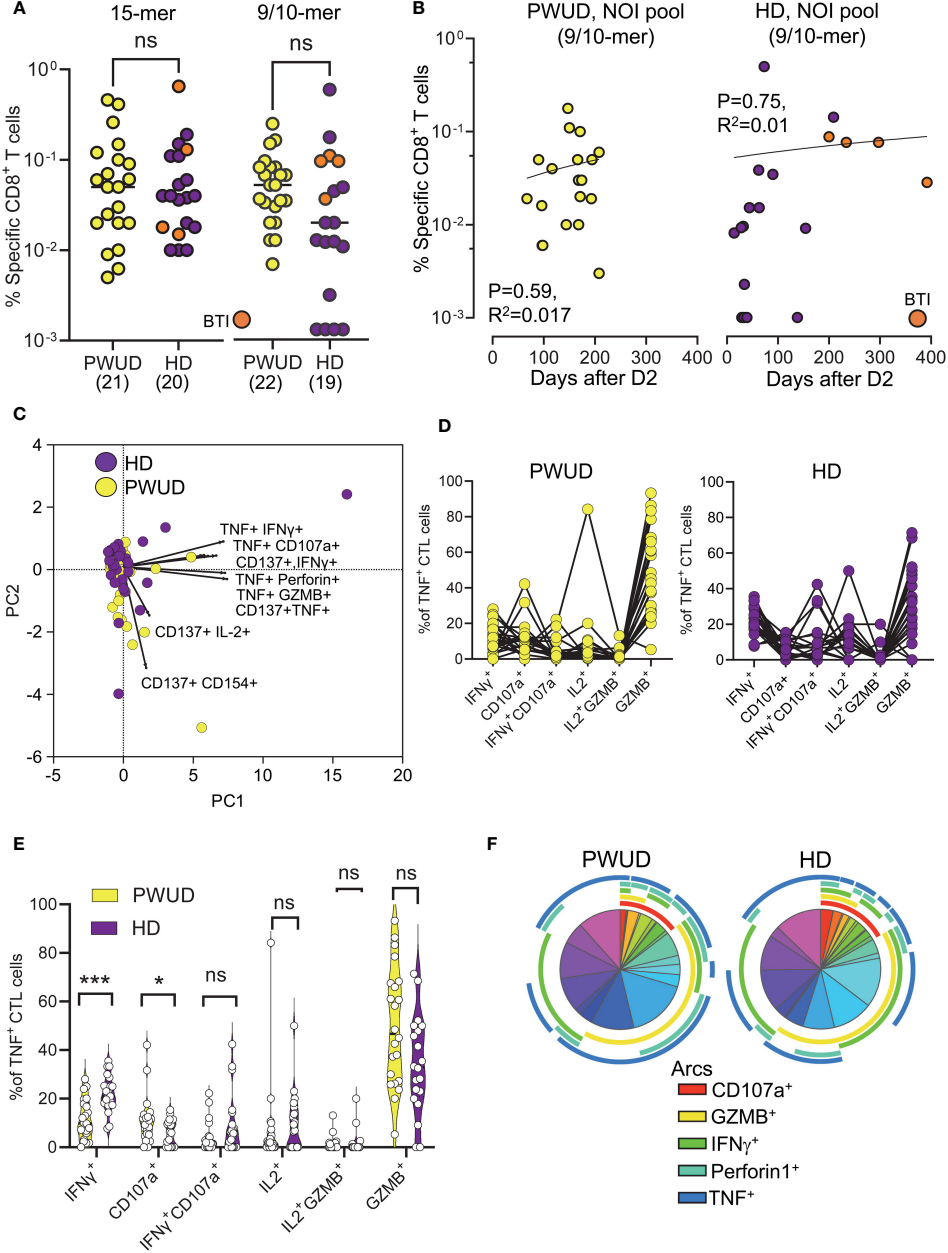
Spike-specific CD8 CTL cell responses in vaccinated donors. Response of CD8 CTL cells (% response − background frequency) quantified by flow cytometry after 18 h stimulation with the Peptivator mix (15-mer) or the NOI pool (9/10-mer). **(A)** Frequency of responder cells gated by Boolean analysis (OR) within the double positive regions (see **Supplementary Figure S2**) for each patient (PWUD vs. HD) and stimulus type (Peptivator mix vs. NOI pool); Mann–Whitney U test; p > 0.05, ns. BTI, breakthrough infection. **(B)** Data from **(A)** are plotted in function of the time after vaccination for both PWUD (left) and HD (right) donors. The correlative trend was not significant. **(C)** PCA analysis and loading plot dimensions for PWUD and HD donors. **(D)** T-cell polyfunctionality in PWUD (left) and HD (right) donors quantified as % of TNF^+^ CD8 CTL cells expressing different marker combinations. **(E)** Violin plots of data from **(D)**; significant differences are shown; Mann–Whitney U test, *p < 0.05, ***p < 0.0001. **(F)** Boolean analysis of the polyfunctional responses of CD8 CTL cells visualized for PWUD and HD donors using SPICE 6. Pie charts represent the median frequency of T-cell-expressing combinations of the indicated markers after stimulation with the Peptivator mix: the arcs indicate the proportion of cells that express CD107 and GZMB (red), CD107 and Perforin (yellow), CD137 (green), IFNγ (light blue), and TNF (blue). The results for PWUD and HD donors are shown on the right and the left, respectively. A detailed list of the populations represented by the pie segments can be found in **Supplementary Table S3**.

**Figure 4:**
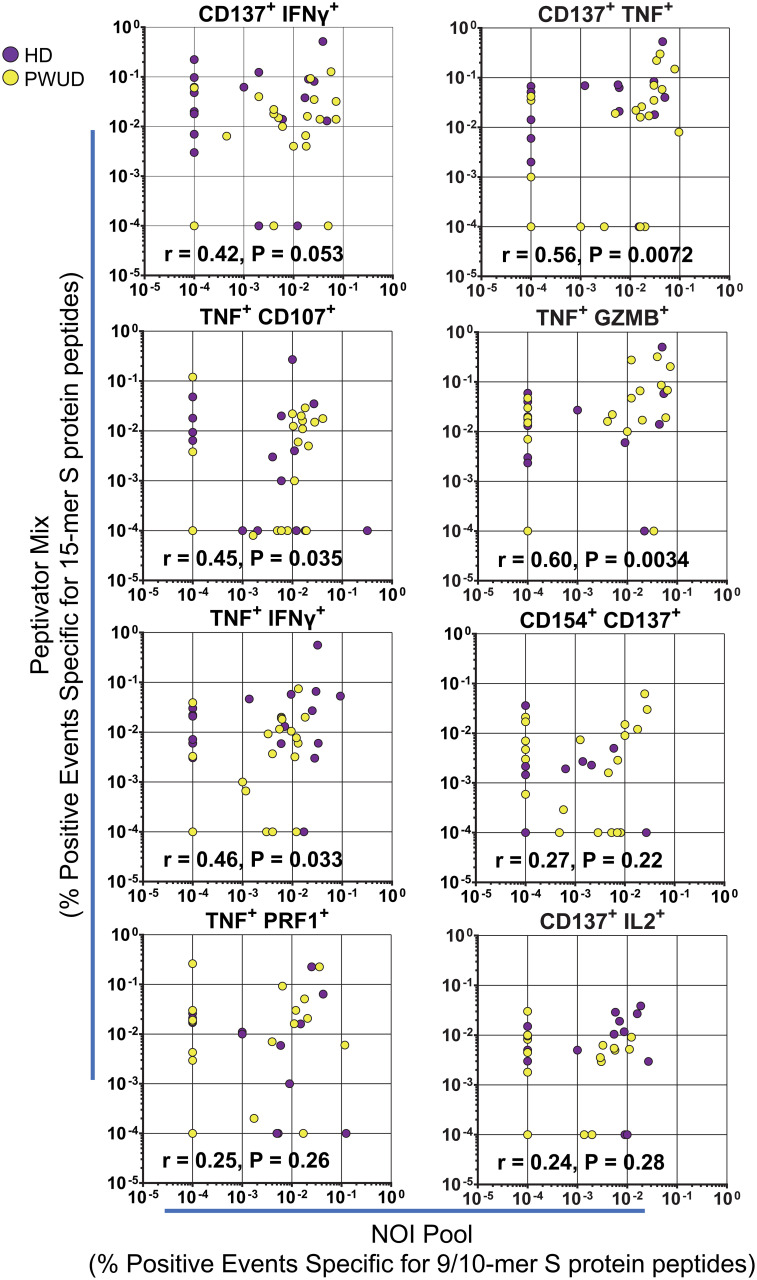
Spike-specific CD8 CTL cell polyfunctionality. Biplots of CD8 CTL cell responses in PWUD and HD donors after stimulation with the Peptivator mix or the NOI pool. The frequencies of CD8 CTL cells that are double positive for CD137^+^ IFNγ^+^, CD137^+^ TNF^+^, TNF^+^ CD107a^+^, TNF^+^ GZMB^+^, TNF^+^ IFNγ^+^, TNF^+^ Perforin^+^, and CD137^+^ IL-2^+^ are presented for each donor from both groups. Spearman’s r correlation and p-values relative to the PWUD cohort are shown.

To investigate the response of CD8^+^ cytotoxic T lymphocytes (CTL), we stimulated the cells with the Peptivator mix or a pool of 23 9/10-mer spike peptides (the NOI pool) that were predicted by the NEC Immune profiler AI tool (see **Supplementary Methods** for peptide list) (43) and experimentally validated (41). We determined the frequency of spike-specific T cells after stimulation with either the Peptivator mix or the NOI pool by measuring CD8 CTL cell responses using the Boolean “Or” gating on the same double positive regions defined for the analysis of the Th cell subset. We found that the frequency of spike-specific CD8 CTL in PWUD and HD donors was not significantly different (**Figure 3A**), remained stable with time (**Figure 3B**), and was characterized by similar response patterns (**Figure 3C**). Likewise, the pattern of CD8 CTL cell polyfunctionality in PWUD was not significantly different from the one observed in healthy controls (**Figure 3D**), an observation further confirmed by Pearson correlation analysis (**Supplementary Figure S4B**), which showed overall similarity between cohorts in terms of immunophenotypic response. A more in-depth analysis of polyfunctionality revealed that among spike-specific (TNF^+^) CTL cells, the fraction of events that were double or triple positive for some markers, including IFNγ, CD107, IL-2, and/or GZMB, was similar to the HD group (**Figure 3E**; 1.8%, 2.0%, 0%, and 47% vs. 5.7%, 6.9%, 0%, and 33% median frequency values for IFNγ^+^ CD107a^+^, IL-2^+^, IL-2^+^ GZMB^+^, and GZMB^+^ populations in PWUD and HD, respectively), but other T-cell populations, such as IFNγ^+^ TNF^+^ CTL cells, appeared to be more frequent in HD (**Figure 3E**; 9.9% vs. 21% Mann–Whitney U test, ***p = 0.001). Conversely, the fraction of degranulating cells expressing TNF (TNF^+^ CD107a^+^ CD8 CTL cells) increased significantly in PWUD (**Figure 3E**; 9.6% vs. 5.9%; Mann–Whitney U test, *p < 0.05). Nevertheless, when we analyzed all permutations using the multiparameter SPICE visualization, we found that the polyfunctional response of the CD8 CTL cell fraction was not significantly different from HD and thus broadly preserved in PWUD (**Figure 3F**). Altogether, these data indicate that PWUD have a normal response to vaccination, but the frequency of some subpopulations differed from the healthy controls. Next, to further investigate the characteristics of the antigenic response to the vaccine in PWUD, we performed a direct comparison of the CD8 CTL cell response to the stimulation with 15-mer and 9/10-mer peptide pools (Peptivator mix vs. NOI pool). Biplots of T-cell responses based on the frequency of cell populations expressing different marker combinations revealed a high degree of linearity and a substantial overlap between the two cohorts in terms of response (**Figure 4**). These results were replicated in a subgroup of donor using a simplified AIM assay (**Supplementary Methods** and data not shown).

### 3.4 Correlation between T-cell reactivity and IgG anti-RBD levels in PWUD

**Figure 5:**
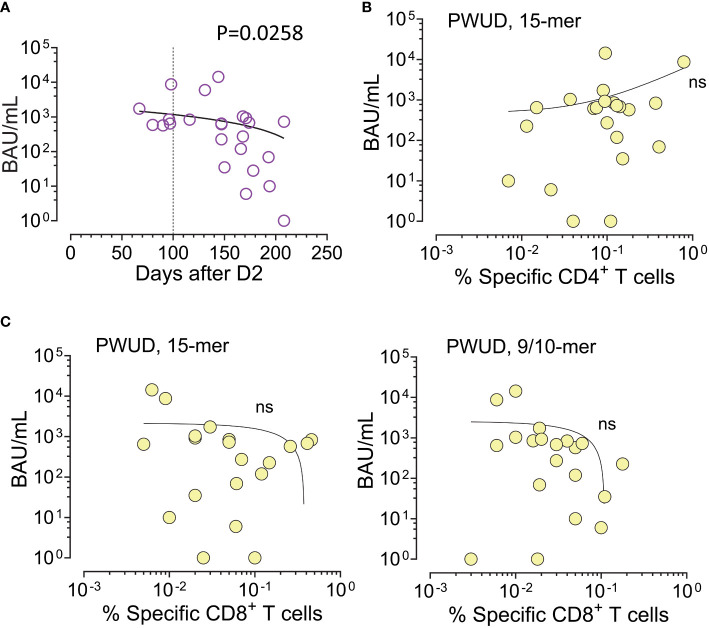
IgG anti-RBD and spike-specific T-cell response after dose 2 in PWUD. **(A)** Scatter plot of serum IgG anti-RBD values (BAU/mL) in function of the number of days post-vaccination (D2). Spearman’s r = −0449; p = 0.0258. **(B, C)** Scatter plot of serum IgG anti-RBD levels vs. T-cell response defined as the frequency of vaccine-specific CD4 Th **(A)** and CD8 CTL **(B)** cells (increase over unstimulated background). T-cell response data shown here are equivalent to the data shown in **Figures 2A**, **3A**. Correlations were not significant.

As observed for the general population, serum IgG anti-RBD level (BAU/mL) decayed with time after vaccination in PWUD (**Figure 5A**). Furthermore, we found no significant correlation between humoral and spike-specific CD4 Th cell response to the Peptivator mix (**Figure 5B**). Similarly, the humoral response did not correlate with CD8 CTL response, notwithstanding the type of stimulation (**Figure 5C**).

## 4 Discussion

In this cross-sectional study, we investigated the T-cell response to SARS-CoV-2 in unvaccinated, SARS-CoV-2 naive people who use drugs (PWUD). Our findings revealed no significant increase in pre-existing T-cell reactivity among PWUD compared to controls. Post-vaccination, the frequency of spike-specific CD4 Th cells in PWUD was comparable to the frequency observed in healthy individuals, but there were some differences between the groups in the frequency of specific cell subpopulations, some of which were higher in the control group, while others, such as cell-expressing cytotoxic markers, were upregulated in the PWUD cohort. Despite these variations, our comprehensive polyfunctionality analysis of spike-specific Th cells showed no significant differences from healthy donor controls in terms of overall response. Similarly, CD8^+^ cytotoxic T lymphocytes (CTL) in PWUD exhibited a frequency of spike-specific responses that was not significantly different from controls. While some differences in CTL subpopulations were noted, the polyfunctionality analysis indicated only minor variations in response distribution, with no significant disparities compared to healthy controls.

Recent studies suggest that a two- or three-dose vaccination regimen provides substantial protection against severe COVID-19 in PWUD, a conclusion that aligns with our observations (38, 50), as the phenotypic differences detected here do not appear to be consequential in terms of vaccine effectiveness against COVID-19 infection or long-term protection in these patients. Our data corroborate these reports, indicating that PWUD exhibit a T-cell response, including polyfunctional activity, comparable to that of healthy controls, which potentially contributes to their protection against severe COVID-19. It is important to note that PWUD are at high risk for a variety of infections including HIV, hepatitis C virus (HCV), hepatitis B virus (HBV), hepatitis A virus (HAV), tetanus, and syphilis (51). Moreover, since many lack stable living arrangements and live outdoor, expose to harsh environments and low temperatures, or are in crowded shelters, PWUD are at a greater risk for secondary bacterial pneumonia (52), influenza, and COVID-19 (11–25) infections. In this regard, it is important to consider that in addition to lifestyle, the chronic abuse of opioids severely compromises both innate and adaptive immune responses through various pathways, signaling cascades, and cytokine networks, and through direct effects on cells, which increase the risk of opportunistic infections (26). Opioids suppress macrophage and T-cell responses through a cascade of anti-inflammatory and anti-autocrine effects that inhibit early pro-inflammatory events, enhance Treg function, block CD4 Th cell proliferation, and downregulate MHC class II expression/antigen presentation (26–29). Previous studies have shown that some transcription factors, such as eomesodermin, play an important role in cytotoxic CD4^+^ T-cell-mediated antiviral immunity in the lungs of severe cases of COVID-19 (53), but the function of these cells in the post-vaccination setting remained unexplored. Our observation of greater cytotoxic marker upregulation in vaccine-specific CD4 Th cells from PWUD donors is therefore of interest and lends support to the recognized role that cytotoxic CD4 Th cells play in antiviral immunity (54).

The serological response of PWUD to vaccines against several pathogens is well documented and varies according to the pathogen. For instance, although the humoral response to vaccination against hepatitis virus, a major health issue for these patients (52), is inadequate as the titers of neutralizing antibodies (NAbs) in PWUD subjects are lower than the general population and boosting would be necessary to help generate protective immunity (51, 55–57), a large body of literature describes vaccines against other viral pathogens, such as HBV (51, 58) and influenza (59–61), as both immunogenic and safe for the prevention of infection. Our data on the humoral response to SARS-CoV-2 vaccination align with previous observations for influenza and hepatitis B virus (HBV) vaccinations, supporting the idea that people who use drugs (PWUD) are capable of developing a protective humoral response against SARS-CoV-2 (38). Furthermore, serum IgG anti-RBD levels in PWUD decreased with time after vaccination but did not correlate with CD4 Th cell response. This observation does not replicate what we observed in other immunocompromised cohorts (42), possibly because of the limited number of subjects analyzed. It is also important to note that high-affinity antibody response may be associated with specific Th cell subsets. For instance, it has been shown that CXCR5^+^ T follicular helper (Tfh) cells are upregulated during the acute phase of SARS-CoV-2 infection, and their frequency directly correlates with IgG anti-spike antibodies (62–64).

Vaccine-induced CD8 CTL cells play a crucial role in the adaptive response to viral infection, and it has been shown that CTL are dysfunctional in severe cases of COVID-19 (65). Thus, the characterization of these cells is particularly relevant in PWUD, as opioids, such as methadone, can decrease effector memory CD8^+^ T cells, without altering the expression of functional markers (30). Our observation of the existence of a CTL polyfunctional response in these patients supports the notion that vaccination helps overcome dysfunction and underscore the importance of booster vaccination in reducing potential severe COVID-19-related outcomes in this high-risk population. Furthermore, our direct comparison of changes in CD8 CTL cell activation markers after stimulation with different peptide pools confirmed a substantial equivalence between PWUD and healthy donors in terms of reactivity. Indeed, the responses of CD8 CTL cells to stimulation with 9–10 amino acid long peptides (NOI pool) was lower than the response to the Peptivator mix (15-mer peptide pools) in some donors from both cohorts, presumably because of an HLA mismatch for some of the peptides that composed the pools. In other donors, CTL response to the NOI pool was not recapitulated by the Peptivator mix, an observation that may likely relate to the fact that 15-mer but not 9/10-mer peptides require trimming for the binding of MHC class I molecules.

In conclusion, we found that mRNA SARS-CoV-2 vaccination stimulates robust polyfunctional T-cell immunity in PWUD despite the reported inhibitory effects that opioids have on the immune response and the detrimental lifestyle and comorbidities that characterized this patient population. This study highlights the need to prioritize booster vaccination to reduce the potential for severe COVID-19-related outcomes and mitigate the spread of infection, which remains a significant health and societal risk for this particularly vulnerable group. Nonetheless, sample size remains a limitation of the current study, and longitudinal follow-up studies of larger cohorts or new vaccination trials will be required to help substantiate our findings.

## Data availability statement

The original contributions presented in the study are included in the article/**Supplementary Materials**, further inquiries can be directed to l.a.munthe@medisin.uio.no.

## Ethics statement

The studies involving humans were approved by Research Ethics Committee Rek SørØst, Norway (Referral numbers of the ethical approvals: REK-SØ 166545 and REK-SØ 135924/COVID-19). The studies were conducted in accordance with the local legislation and institutional requirements. The participants provided their written informed consent to participate in this study.

## Author contributions

LM, JV, and LW designed the study, RS, ST, BM, and TC provided peptide predictions. HK designed the flow cytometry panel. MG, LF, JR, VC, AA, FL-J, and LM analyzed data. MG, LF, JR, and LM prepared Figures. All authors contributed to data analysis and interpretation. KH, GW, and JAJ recruited PWUD donors and provided clinical information. LW administered PWUD recruitment and collected clinical study parameters. LM wrote the first draft of the paper. All authors contributed to the article and approved the submitted version.

## References

[1] Reece AS. Clinical implications of addiction related immunosuppression. J Infection. (2008) 56(6):437–45. doi: 10.1016/j.jinf.2008.03.003 18440646

[2] Thompson RG Wall MM Greenstein E Grant BF Hasin DS. Substance-use disorders and poverty as prospective predictors of first-time homelessness in the United States. Am J Public Health (2013) 103(S2):S282–S8. doi: 10.2105/AJPH.2013.301302 PMC3865876 24148043

[3] Dickey B Normand SL Weiss RD Drake RE Azeni H. Medical morbidity, mental illness, and substance use disorders. Psychiatr Serv. (2002) 53(7):861–7. doi: 10.1176/appi.ps.53.7.861 12096170

[4] Batki SL Meszaros ZS Strutynski K Dimmock JA Leontieva L Ploutz-Snyder R. Medical comorbidity in patients with schizophrenia and alcohol dependence. Schizophr Res (2009) 107(2-3):139–46. doi: 10.1016/j.schres.2008.10.016 PMC2649875 19022627

[5] Saha S Chant D McGrath J. A systematic review of mortality in schizophrenia: is the differential mortality gap worsening over time? Arch Gen Psychiatry (2007) 64(10):1123–31. doi: 10.1001/archpsyc.64.10.1123 17909124

[6] Nordentoft M Wahlbeck K Hällgren J Westman J Osby U Alinaghizadeh H. Excess mortality, causes of death and life expectancy in 270,770 patients with recent onset of mental disorders in Denmark, Finland and Sweden. PloS One (2013) 8(1):e55176. doi: 10.1371/journal.pone.0055176 23372832 PMC3555866

[7] Olfson M Gerhard T Huang C Crystal S Stroup TS. Premature mortality among adults with Schizophrenia in the United States. JAMA Psychiatry (2015) 72(12):1172–81. doi: 10.1001/jamapsychiatry.2015.1737 26509694

[8] Heiberg IH Jacobsen BK Nesvåg R Bramness JG Reichborn-Kjennerud T Næss Ø. Total and cause-specific standardized mortality ratios in patients with schizophrenia and/or substance use disorder. PloS One (2018) 13(8):e0202028. doi: 10.1371/journal.pone.0202028 30138449 PMC6107156

[9] Hjorthøj C Stürup AE McGrath JJ Nordentoft M. Years of potential life lost and life expectancy in schizophrenia: a systematic review and meta-analysis. Lancet Psychiatry (2017) 4(4):295–301. doi: 10.1016/S2215-0366(17)30078-0 28237639

[10] Chang CK Hayes RD Perera G Broadbent MT Fernandes AC Lee WE. Life expectancy at birth for people with serious mental illness and other major disorders from a secondary mental health care case register in London. PloS One (2011) 6(5):e19590. doi: 10.1371/journal.pone.0019590 21611123 PMC3097201

[11] Baggett TP Keyes H Sporn N Gaeta JM. Prevalence of SARS-CoV-2 infection in residents of a large homeless shelter in Boston. JAMA-J Am Med Assoc (2020) 323(21):2191–2. doi: 10.1001/jama.2020.6887 PMC7186911 32338732

[12] Barocas JA. Business not as usual - Covid-19 vaccination in persons with substance use disorders. N Engl J Med (2021) 384(2):e6. doi: 10.1056/NEJMpv2035709 33378604

[13] Dubey MJ Ghosh R Chatterjee S Biswas P Chatterjee S Dubey S. COVID-19 and addiction. Diabetes Metab Syndr (2020) 14(5):817–23. doi: 10.1016/j.dsx.2020.06.008 PMC7282772 32540735

[14] Melamed OC Hauck TS Buckley L Selby P Mulsant BH. COVID-19 and persons with substance use disorders: Inequities and mitigation strategies. Subst abuse: Off Publ Assoc Med Educ Res Subst Abuse. (2020) 41(3):286–91. doi: 10.1080/08897077.2020.1784363 32697172

[15] Ornell F Moura HF Scherer JN Pechansky F Kessler FHP von Diemen L. The COVID-19 pandemic and its impact on substance use: Implications for prevention and treatment. Psychiatry Res (2020) 289:113096. doi: 10.1016/j.psychres.2020.113096 32405115 PMC7219362

[16] Alexander GC Stoller KB Haffajee RL Saloner B. An epidemic in the midst of a pandemic: opioid use disorder and COVID-19. Ann Intern Med (2020) 173(1):57–8. doi: 10.7326/M20-1141 PMC7138407 32240283

[17] Volkow ND. Collision of the COVID-19 and addiction epidemics. Ann Intern Med (2020) 173(1):61–2. doi: 10.7326/M20-1212 PMC7138334 32240293

[18] Spagnolo PA Montemitro C Leggio L. New challenges in addiction medicine: COVID-19 infection in patients with alcohol and substance use disorders—The perfect storm. Am J Psychiatry (2020) 177(9):805–7. doi: 10.1176/appi.ajp.2020.20040417 PMC9366241 32660296

[19] Wen H Barnett ML Saloner B. Clinical risk factors for COVID-19 among people with substance use disorders. Psychiatr Serv. (2020) 71(12):1308. doi: 10.1176/appi.ps.202000215 33019859

[20] Wei Y Shah R. Substance use disorder in the COVID-19 pandemic: A systematic review of vulnerabilities and complications. Pharmaceuticals (2020) 13(7):155. doi: 10.3390/ph13070155 32708495 PMC7407364

[21] Marsden J Darke S Hall W Hickman M Holmes J Humphreys K. Mitigating and learning from the impact of COVID-19 infection on addictive disorders. Addiction (2020) 115(6):1007–10. doi: 10.1111/add.15080 PMC9364227 32250482

[22] Wang QQ Kaelber DC Xu R Volkow ND. COVID-19 risk and outcomes in patients with substance use disorders: analyses from electronic health records in the United States. Mol Psychiatry (2021) 26(1):30–9. doi: 10.1038/s41380-020-00880-7 PMC7488216 32929211

[23] Vai B Mazza MG Colli CD Foiselle M Allen B Benedetti F. Mental disorders and risk of COVID-19-related mortality, hospitalisation, and intensive care unit admission: a systematic review and meta-analysis. Lancet Psychiatry (2021) 8(9):797–812. doi: 10.1016/S2215-0366(21)00232-7 34274033 PMC8285121

[24] Catalan A Aymerich C Bilbao A Pedruzo B Perez JL Aranguren N. Psychosis and substance abuse increase the COVID-19 mortality risk. psychol Med (2022) 9:4236–44. doi: 10.1017/S0033291722000976 PMC9114752 35410632

[25] Hashemi-Shahri SM Tabatabaei SMN Ansari-Moghaddam A Mohammadi M Okati-Aliabad H Tabatabaei SM. Epidemiological and clinical risk factors related to severe COVID-19 in Iran: a multi-center study. BMC Infect diseases. (2022) 22(1):11. doi: 10.1186/s12879-022-07165-0 35197013 PMC8864589

[26] Roy S Ninkovic J Banerjee S Charboneau RG Das S Dutta R. Opioid drug abuse and modulation of immune function: consequences in the susceptibility to opportunistic infections. J Neuroimmune Pharmacol (2011) 6(4):442–65. doi: 10.1007/s11481-011-9292-5 PMC3601186 21789507

[27] Hansen W Luppus S Barthel R Chang DI Broemstrup J Zwarg T. Heroin-assisted treatment of heroin-addicted patients normalizes regulatory T cells but does not restore CD4+ T cell proliferation. Addict Biol (2021) 26(4):e12998. doi: 10.1111/adb.12998 33336491

[28] Rivera-Amill V Kumar R Noel RJ Jr. Garcia Y Rodriguez IV Martinez M. Short communication: Lack of immune response in rapid progressor morphine-dependent and SIV/SHIV-infected rhesus macaques is correlated with downregulation of TH1 cytokines. AIDS Res Hum Retroviruses (2010) 26(8):919–22. doi: 10.1089/aid.2010.0012 PMC2957625 20672973

[29] Nugent AL Houghtling RA Bayer BM. Morphine suppresses MHC-II expression on circulating B lymphocytes via activation of the HPA. J Neuroimmune Pharmacol (2011) 6(1):130–41. doi: 10.1007/s11481-010-9218-7 PMC3022947 20440572

[30] Mazahery C Benson BL Cruz-Lebrón A Levine AD. Chronic methadone use alters the CD8(+) T cell phenotype *in vivo* and modulates its responsiveness *ex vivo* to opioid receptor and TCR stimuli. J Immunol (2020) 204(5):1188–200. doi: 10.4049/jimmunol.1900862 PMC7757720 31969385

[31] Swadling L Diniz MO Schmidt NM Amin OE Chandran A Shaw E. Pre-existing polymerase-specific T cells expand in abortive seronegative SARS-CoV-2. Nature (2022) 601(7891):110–7. doi: 10.1038/s41586-021-04186-8 PMC8732273 34758478

[32] Woldemeskel BA Garliss CC Blankson JN. SARS-CoV-2 mRNA vaccines induce broad CD4+ T cell responses that recognize SARS-CoV-2 variants and HCoV-NL63. J Clin Invest. (2021) 131(10):e149335. doi: 10.1172/JCI149335 33822770 PMC8121504

[33] Rydyznski Moderbacher C Ramirez SI Dan JM Grifoni A Hastie KM Weiskopf D. Antigen-specific adaptive immunity to SARS-CoV-2 in acute COVID-19 and associations with age and disease severity. Cell (2020) 183(4):996–1012.e19. doi: 10.1016/j.cell.2020.09.038 33010815 PMC7494270

[34] Tan AT Linster M Tan CW Le Bert N Chia WN Kunasegaran K. Early induction of functional SARS-CoV-2-specific T cells associates with rapid viral clearance and mild disease in COVID-19 patients. Cell Rep (2021) 34(6):108728. doi: 10.1016/j.celrep.2021.108728 33516277 PMC7826084

[35] McMahan K Yu J Mercado NB Loos C Tostanoski LH Chandrashekar A. Correlates of protection against SARS-CoV-2 in rhesus macaques. Nature (2021) 590(7847):630–4. doi: 10.1038/s41586-020-03041-6 PMC7906955 33276369

[36] Swadling L Diniz MO Schmidt NM Amin OE Chandran A Shaw E. Pre-existing polymerase-specific T cells expand in abortive seronegative SARS-CoV-2. Nature (2021) 601(7891):110–7. doi: 10.1101/2021.06.26.21259239 PMC8732273 34758478

[37] Kared H Redd AD Bloch EM Bonny TS Sumatoh H Kairi F. SARS-CoV-2-specific CD8+ T cell responses in convalescent COVID-19 individuals. J Clin Invest (2021) 131(5):e145476. doi: 10.1172/JCI145476 33427749 PMC7919723

[38] Huang C Wei Y Yan VKC Ye X Kang W Yiu HHE. Vaccine effectiveness of BNT162b2 and CoronaVac against SARS-CoV-2 omicron infection and related hospital admission among people with substance use disorder in Hong Kong: a matched case-control study. Lancet Psychiatry (2023) 10(6):403–13. doi: 10.1016/S2215-0366(23)00111-6 PMC10191606 37141907

[39] Wüsthoff L Lund-Johansen F Henriksen K Wildendahl G Jacobsen J-A Gomes L. Seroprevalence of SARS-CoV-2 and humoral immune responses to mRNA vaccines among people who use drugs - In the light of tailored mitigating strategies. Res Square (2023). doi: 10.21203/rs.3.rs-2939683/v1 PMC11186241 38890611

[40] Tran TT Vaage EB Mehta A Chopra A Tietze L Kolderup A. Titers of antibodies against ancestral SARS-CoV-2 correlate with levels of neutralizing antibodies to multiple variants. NPJ Vaccines (2022) 7(1):174. doi: 10.1038/s41541-022-00586-7 36585405 PMC9801350

[41] Federico L Malone B Tennøe S Chaban V Osen JR Gainullin M. Experimental validation of immunogenic SARS-CoV-2 T cell epitopes identified by artificial intelligence. Front Immunol (2023) 14. doi: 10.3389/fimmu.2023.1265044 PMC10691274 38045681

[42] Federico L Tvedt THA Gainullin M Osen JR Chaban V Lund KP. Robust spike-specific CD4+ and CD8+ T cell responses in SARS-CoV-2 vaccinated hematopoietic cell transplantation recipients: a prospective, cohort study. Front Immunol (2023) 14. doi: 10.3389/fimmu.2023.1210899 PMC10369799 37503339

[43] Malone B Simovski B Moliné C Cheng J Gheorghe M Fontenelle H. Artificial intelligence predicts the immunogenic landscape of SARS-CoV-2 leading to universal blueprints for vaccine designs. Sci Rep (2020) 10(1):22375. doi: 10.1038/s41598-020-78758-5 33361777 PMC7758335

[44] Tarke A Sidney J Methot N Yu ED Zhang Y Dan JM. Impact of SARS-CoV-2 variants on the total CD4(+) and CD8(+) T cell reactivity in infected or vaccinated individuals. Cell Rep Med (2021) 2(7):100355. doi: 10.1016/j.xcrm.2021.100355 34230917 PMC8249675

[45] Reiss S Baxter AE Cirelli KM Dan JM Morou A Daigneault A. Comparative analysis of activation induced marker (AIM) assays for sensitive identification of antigen-specific CD4 T cells. PloS One (2017) 12(10):e0186998. doi: 10.1371/journal.pone.0186998 29065175 PMC5655442

[46] Braun J Loyal L Frentsch M Wendisch D Georg P Kurth F. SARS-CoV-2-reactive T cells in healthy donors and patients with COVID-19. Nature (2020) 587(7833):270–4. doi: 10.1038/s41586-020-2598-9 32726801

[47] Altosole T Rotta G Uras CRM Bornheimer SJ Fenoglio D. An optimized flow cytometry protocol for simultaneous detection of T cell activation induced markers and intracellular cytokines: Application to SARS-CoV-2 immune individuals. J Immunol Methods (2023) 515:113443. doi: 10.1016/j.jim.2023.113443 36842524 PMC9957341

[48] Roederer M Nozzi JL Nason MC. SPICE: Exploration and analysis of post-cytometric complex multivariate datasets. Cytometry Part A (2011) 79A(2):167–74. doi: 10.1002/cyto.a.21015 PMC3072288 21265010

[49] . Available at: https://niaid.github.io/spice.

[50] Wang L Wang Q Davis PB Volkow ND Xu R. Increased risk for COVID-19 breakthrough infection in fully vaccinated patients with substance use disorders in the United States between December 2020 and August 2021. World Psychiatry (2022) 21(1):124–32. doi: 10.1002/wps.20921 PMC8661963 34612005

[51] Baral S Sherman SG Millson P Beyrer C. Vaccine immunogenicity in injecting drug users: a systematic review. Lancet Infect Diseases. (2007) 7(10):667–74. doi: 10.1016/S1473-3099(07)70237-2 17897609

[52] Quaglio G Lugoboni F Mezzelani P Des Jarlais DC Lechi A. Hepatitis vaccination among drug users. Vaccine (2006) 24(15):2702–9. doi: 10.1016/j.vaccine.2005.12.045 16436307

[53] Kaneko N Boucau J Kuo H-H Perugino C Mahajan VS Farmer JR. Temporal changes in T cell subsets and expansion of cytotoxic CD4+ T cells in the lungs in severe COVID-19. Clin Immunol (2022) 237:108991. doi: 10.1016/j.clim.2022.108991 35364330 PMC8961941

[54] Marshall NB Swain SL. Cytotoxic CD4 T cells in antiviral immunity. J BioMed Biotechnol (2011) 2011:954602. doi: 10.1155/2011/954602 22174559 PMC3228492

[55] Lugoboni F Migliozzi S Mezzelani P Pajusco B Ceravolo R Quaglio G. Progressive decrease of hepatitis B in a cohort of drug users followed over a period of 15 years: The impact of anti-HBV vaccination. Scandinavian J Infect Diseases. (2004) 36(2):131–3. doi: 10.1080/00365540310018833 15061668

[56] Lemon SM Thomas DL. Vaccines to prevent viral hepatitis. New Engl J Med (1997) 336(3):196–204. doi: 10.1056/NEJM199701163360307 8988900

[57] Quaglio G Pajusco B Civitelli P Migliozzi S Des Jarlais DC Romanò L. Immunogenicity, reactogenicity and adherence with hepatitis A vaccination among drug users. Drug Alcohol dependence. (2004) 74(1):85–8. doi: 10.1016/j.drugalcdep.2003.12.001 15072811

[58] Schalm SW Heytink RA Mannaerts H Vreugdenhil A. Immune response to hepatitis B vaccine in drug addicts. J Infection. (1983) 7:41–5. doi: 10.1016/S0163-4453(83)96603-3 6674369

[59] Amendola A Boschini A Colzani D Anselmi G Oltolina A Zucconi R. Influenza vaccination of HIV-1-positive and HIV-1-negative former intravenous drug users. J Med virology. (2001) 65(4):644–8. doi: 10.1002/jmv.2085 11745926

[60] Iorio AM Alatri A Francisci D Preziosi R Neri M Donatelli I. Immunogenicity of influenza vaccine (1993–1994 winter season) in HIV-seropositive and-seronegative ex-intravenous drug users. Vaccine (1997) 15(1):97–102. doi: 10.1016/S0264-410X(96)00057-6 9041673

[61] Moroz E Albrecht RA Aden B Beeder AB Yuan J García-Sastre A. Active opioid use does not attenuate the humoral responses to inactivated influenza vaccine. Vaccine (2016) 34(11):1363–9. doi: 10.1016/j.vaccine.2016.01.051 PMC4780674 26859239

[62] Juno JA Tan HX Lee WS Reynaldi A Kelly HG Wragg K. Humoral and circulating follicular helper T cell responses in recovered patients with COVID-19. Nat Med (2020) 26(9):1428–34. doi: 10.1038/s41591-020-0995-0 32661393

[63] He R Zheng X Zhang J Liu B Wang Q Wu Q. SARS-CoV-2 spike-specific T(FH) cells exhibit unique responses in infected and vaccinated individuals. Signal Transduct Target Ther (2023) 8(1):393. doi: 10.1038/s41392-023-01650-x 37802996 PMC10558553

[64] Zhang J Wu Q Liu Z Wang Q Wu J Hu Y. Spike-specific circulating T follicular helper cell and cross-neutralizing antibody responses in COVID-19-convalescent individuals. Nat Microbiol (2021) 6(1):51–8. doi: 10.1038/s41564-020-00824-5 33199863

[65] Gozzi-Silva SC Oliveira LM Alberca RW Pereira NZ Yoshikawa FS Pietrobon AJ. Generation of cytotoxic T cells and dysfunctional CD8 T cells in severe COVID-19 patients. Cells (2022) 11(21):3359. doi: 10.3390/cells11213359 36359755 PMC9659290

