# Supplementary material for: People who use drugs show no increase in pre-existing T-cell cross-reactivity toward SARS-CoV-2 but develop a normal polyfunctional T-cell response after standard mRNA vaccination
Source: Front Immunol. 2024 Jan 17;14:1235210. doi: 10.3389/fimmu.2023.1235210 (PMC10827924; doi:10.3389/fimmu.2023.1235210)
Supplement: Supplementary file 1 [file Presentation_1.pdf]

People who use drugs show no increase in pre-existing T cell cross-reactivity toward SARS-CoV-2 but develop a normal polyfunctional T cell response after standard mRNA vaccination.

Murat Gainullin[1,2,3]*, Lorenzo Federico[1,3]*, Julie Røkke Osen[1,3], Viktoriia Chaban[1,3], Hassen Kared[1,3], Amin Alirezaylavasani[1,3], Fridtjof Lund-Johansen[3,5,6], Gull Wildendahl[4], Jon-Aksel Jacobsen[4], Hina Sarwar Anjum[4], Richard Stratford[2], Simen Tennøe[2], Brandon Malone[2], Trevor Clancy[2], John T. Vaage[3,6], Kathleen Henriksen[4,7], Linda Wüsthoff[8, 9]*, Ludvig A. Munthe[1,3]*

* Contributed equally

Corresponding authors: Linda Wüsthoff, linwus@ous-hf.no and Ludvig A. Munthe, l.a.munthe@medisin.uio.no

Supplementary Data and Figures

Summary:

Supplementary Methods

- Simplified antibody panel for flow cytometry.
- 9/10-mer peptides used for pre-vaccination study.
- 9-10-mer spike peptides used for post-vaccination study.

Supplementary Figures and Tables

- **Supplementary Figure S1**. T cell IFNγ production in response to SARS-CoV-2 peptides in
- unvaccinated, non-infected individuals.
- **Supplementary Figure S2**. Flow cytometry gating strategy.
- **Supplementary Figure S3.** Spike-specific CD4+ T cell responses in vaccinated donors.
- **Supplementary Figure S4**. Correlation pattern of AIM combinations for CD4+ or CD8+ T cells.
- **Supplementary Table S1**. Patient demographics.
- **Supplementary Table S2**. Clinical information by patient.
- **Supplementary Table S3**. T cell subpopulations (SPICE categories).

Supplementary Methods

Simplified antibody panel for flow cytometry.

Further replicate assays were performed as shown in the Method section using a simplified flow cytometry staining panel that included the following antibodies: BV605 anti-human CD3 (Clone SK7; BD Biosciences, # 563219), PerCP-Cy5.5 anti-human CD4 (Clone OKT4; Biolegend, # 317428), and Alexa Fluor 488 anti-human CD8 (Clone OKT8; Invitrogen, # 53-0086-42), APC anti-human CD137 (Clone 4B4-1; BD Biosciences, # 550890), BV711 anti-human CD40L (Clone 24-31; Biolegend, # 310837), PE anti-human IFNγ (Clone 4S.B3; Biolegend, # 502509), and BV421 anti-human TNFA (Clone MAb11; BD Biosciences, # 562783).

9/10-mer peptides used for pre-vaccination study.

The following peptides were used for the pre-vaccination studies (Peptide sequence, Protein, Top HLA restriction): TRFASVYAW, Spike, HLA-C*07:01/02; AHFPREGVF, Spike, HLA-C*07:01/02; GTHWFVTQR, Spike, HLA-A*03:01; FVSNGTHWFV, Spike, HLA-A*02:01; NSFTRGVYY, Spike, HLA-A*01:01, HLA-A*03:01, HLA-C*07:01/02; RLITGRLQSL, Spike, HLA-A*02:01, HLA-B*08:01; IPTNFTISV, Spike, HLA-B*07:02, HLA-C*03:03; RAAEIRASA, Spike, HLA-B*07:02, HLA-C*03:03; IAIVMVTIM, Spike, HLA-C*03:03; YLQPRTFLL, Spike, HLA-A*01:01, HLA-A*2:01, HLA-A*23:01, HLA-A*29:02, HLA-C*07:01/02; WTAGAAAYY, Spike, HLA-A*01:01, HLA-C*07:01; LPIGINITRF, Spike, HLA-B*07:02; YTNSFTRGVY, Spike, HLA-A*01:01; SPRRARSVA, Spike, HLA-B*07:02; LPPAYTNSF, Spike, HLA-B*07:02, HLA-B*08:01, HLA-C*03:03; SIIAYTMSL, Spike, HLA-A*2:01, HLA-C*07:01/02; ASFSTFKCY, Spike, HLA-A*01:01; EMIAQYTSAL, Spike, HLA-B*15:01/02; YQPYRVVVL, Spike, HLA-A*2:01, HLA-A*23:01, HLA-B*15:01/02, HLA-C*07:01/02; KVGGNYNYLY, Spike, HLA-A*01:01; WTAGAAAYYV, Spike, HLA-A*01:01; FKNLREFVF, Spike, HLA-C*07:01/02; GAAAYYVGY, Spike, HLA-A*01:01; MAYRFNGIG, Spike, HLA-C*03:03; TPINLVRDL, Spike, HLA-B*07:02; FVFKNIDGYF, Spike, HLA-A*23:01, HLA-A*29:02; KTSVDCTMY, Spike, HLA-A*01:01; FVFKNIDGY, Spike, HLA-A*01:01, HLA-C*03:03, HLA-C*07:01/02; TLKSFTVEK, Spike, HLA-A*03:01; GEVFNATRF, Spike, HLA-B*15:01/02, HLA-B*40:01, HLA-B*44:02; FPREGVFVSN, Spike, HLA-B*07:02; ESIVRFPNI, Spike, HLA-B*08:01; AEIRASANL, Spike, HLA-B*15:01/02, HLA-B*40:01, HLA-B*44:02; TRFQTLLAL, Spike, HLA-C*04:01, HLA-C*07:01/02; IPFAMQMAY, Spike, HLA-A*29:02, HLA-B*07:02, HLA-B*15:02, HLA-C*03:03, HLA-C*07:01/02 ; SNIIRGWIF, Spike, HLA-A*23:01; DKVFRSSVL, Spike, HLA-B*08:01; IKWPWYIWL, Spike, HLA-C*07:01/02; TLLALHRSY, Spike, HLA-A*03:01, HLA-A*29:02, HLA-B*15:01/02; VYYPDKVFRS, Spike, HLA-C*07:01/02; FAMQMAYRF, Spike, HLA-A*23:01, HLA-A*29:02, HLA-B*15:02, HLA-C*03:03, HLA-C*04:01, HLA-C*07:01/02 ; LPFNDGVYF, Spike, HLA-A*29:02, HLA-B*07:02, HLA-B*15:02, HLA-C*03:03, HLA-C*04:01, HLA-C*07:01/02; RGWIFGTTL, Spike, HLA-C*03:03, HLA-C*07:01/02; FLYLYALVYF, Orf3a, HLA-A*23:01; SINFVRIIM, Orf3a, HLA-B*07:02; LLYDANYFL, Orf3a, HLA-A*02:01, HLA-A*29:02, HLA-A*23:01, HLA-C*03:03, HLA-C*04:01, HLA-C*07:01/02 ; IIMRLWLCWK, Orf3a, HLA-A*03:0; LEAPFLYLY, Orf3a, HLA-A*01:01, HLA-A*29:02, HLA-B*15:01/02, HLA-B*40:01, HLA-B*44:02 ; LEAPFLYLYA, Orf3a, HLA-B*44:02; APFLYLYAL, Orf3a, HLA-B*07:02, HLA-B*08:01, HLA-C*03:03; APISAMVRM, Orf1ab, HLA-B*07:02, HLA-B*15:02,

HLA-C*03:03; TVAYFNMVY, Orf1ab, HLA-A*01:01, HLA-C*03:03; GLAAIMQLFF, Orf1ab, HLA-A*29:02; REVRTIKVF, Orf1ab, HLA-B*15:01/02, HLA-B*40:01, HLA-B*44:02; RTIKGTHHW, Orf1ab, HLA-A*23:01, HLA-A*29:02, HLA-B*15:01/02, HLA-B*44:02, HLA-C*07:01/02; FHLDGEVITF, Orf1ab, HLA-A*01:01, HLA-A*02:01, HLA-A*23:01, HLA-C*04:01, HLA-C*07:01/02 ; MSYEDQDALF, Orf1ab, HLA-A*01:01, HLA-A*23:01, HLA-A*29:02, HLA-B*15:01/02, HLA-C*04:01; VQMAPISAM, Orf1ab, HLA-A*02:01, HLA-B*07:02, HLA-B*15:01/02, HLA-B*40:01, HLA-C*03:03, HLA-C*04:01, HLA-C*07:01/02 ; WLIINLVQM, Orf1ab, HLA-A*02:01, HLA-C*03:03; MGIIAMSAF, Orf1ab, HLA-B*15:01/02, HLA-C*03:03; ALYNKYKYF, Orf1ab, HLA-A*23:01, HLA-A*29:02, HLA-B*08:01, HLA-B*15:01/02, HLA-C*04:01, HLA-C*07:01/02 ; RARTVAGVSI, Orf1ab, HLA-B*07:02; YEDQDALFAY, Orf1ab, HLA-A*01:01; AIMQLFFSYF, Orf1ab, HLA-A*23:01, HLA-A*29:02, HLA-B*15:01; VVRQCSGVTF, Orf1ab, HLA-B*15:01/02; TQYNRYLALY, Orf1ab, HLA-A*29:02, HLA-B*15:01/02; KHFYWFFSNY, Orf1ab, HLA-A*29:02; KFYGGWHNM, Orf1ab, HLA-A*23:01, HLA-A*29:02, HLA-C*03:03, HLA-B*15:01/02, HLA-C*04:01, HLA-C*07:01/02 ; MMFVKHKHA, Orf1ab, HLA-B*08:01; LFFSYFAVHF, Orf1ab, HLA-A*23:01; IAMSAFAMM, Orf1ab, HLA-B*15:02; APISAMVRMY, Orf1ab, HLA-B*07:02, HLA-B*15:02; MPASWVMRIM, Orf1ab, HLA-B*07:02; YMPASWVMRI, Orf1ab, HLA-A*02:01; AMSAFAMMF, Orf1ab, HLA-A*23:01, HLA-A*29:02, HLA-B*15:01/02, HLA-C*04:01, HLA-C*07:02; HFYWFFSNY, Orf1ab, HLA-A*23:01, HLA-A*29:02, HLA-C*07:01/02; GEVITFDNL, Orf1ab, HLA-B*40:01, HLA-B*44:02; VYYTSNPTTF, Orf1ab, HLA-C*07:01/02, HLA-A*23:01, HLA-A*29:02, HLA-C*04:01 ; LPFAMGIIAM, Orf1ab, HLA-B*07:02; LNKEMYLKL, Orf1ab, HLA-B*08:01; FLLNKEMYL, Orf1ab, HLA-A*02:01, HLA-B*08:01, HLA-C*03:03, HLA-C*04:01, HLA-C*07:01/02; LLPSLATVAY, Orf1ab, HLA-A*01:01, HLA-A*29:02, HLA-B*15:01/02 ; AIMQLFFSY, Orf1ab, HLA-A*03:01, HLA-A*29:02, HLA-B*15:01/02; DMVDTSLSGF, Orf1ab, HLA-B*15:01/02; TQYNRYLAL, Orf1ab, HLA-B*08:01, HLA-C*03:03, HLA-B*15:01/02, HLA-B*40:01, HLA-C*03:03, HLA-C*04:01, HLA-C*07:01/02 ; MPASWVMRI, Orf1ab, HLA-B*07:02, HLA-C*03:03; MVMFTPLVPF, Orf1ab, HLA-B*15:01/02, HLA-A*29:02, HLA-A*23:01; YLKLRSDVL, Orf1ab, HLA-B*08:01, HLA-C*03:03; MNLKYAISA, Orf1ab, HLA-B*08:01; NMVYMPASW, Orf1ab, HLA-A*23:01; TQMNLKYAI, Orf1ab, HLA-B*40:01; VMFTPLVPF, Orf1ab, HLA-A*23:01, HLA-A*29:02, HLA-C*03:03, HLA-B*15:01/02; STKHFYWFF, Orf1ab, HLA-A*29:02; WLMWLIINL, Orf1ab, HLA-A*02:01; AMMFVKHKH, Orf1ab, HLA-A*03:01; SWVMRIMTW, Orf1ab, HLA-A*23:01, HLA-C*07:02; FAYTKRNVI, Orf1ab, HLA-B*07:02, HLA-B*08:01, HLA-C*03:03, HLA-C*04:01, HLA-C*07:01/02 ; HFISNSWLM, Orf1ab, HLA-A*23:01, HLA-A*29:02, HLA-C*04:01, HLA-C*07:01/02; MFVKHKHAF, Orf1ab, HLA-A*23:01, HLA-A*29:02, HLA-B*08:01, HLA-B*15:01/02, HLA-C*04:01, HLA-C*07:01/02 ; ILTALRLCAY, E, HLA-A*29:02; SLVKPSFYVY, E, HLA-A*01:01, HLA-A*03:01, HLA-A*29:02, HLA-B*15:01/02.

9-10-mer spike peptides used for post-vaccination study (The NOI pool).

All peptides used for post-vaccination study derived from the spike protein (Peptide sequence, Top HLA restriction)YTNSFTRGVY, HLA-A*01:01; YLQPRTFLL, HLA-A*01:01/HLA-A*2:01/promiscuous; RLITGRLQSL, HLA-A*02:01/HLA-B*08:01; SPRRARSVA, HLA-B*07:02; GTHWFVTQR, HLA-A*03:01; GAAAYYVGY, HLA-A*01:01; ASFSTFKCY, HLA-A*01:01; KTSVDCTMY, HLA-A*01:01; KVGGNYNYLY, HLA-A*01:01; NSFTRGVYY, HLA-A*01:01/HLA-A*03:01/HLA-C*07:01/02; FVFKNIDGY, HLA-A*01:01/HLA-C*03:03/HLA-

C*07:01/02; WTAGAAAYY, HLA-A*01:01/HLA-C*07:01; FVSNGTHWFV, HLA-A*02:01; SIIAYTMSL, HLA-A*2:01/promiscuous; TPINLVRDL, HLA-B*07:02; LPIGINITRF, HLA-B*07:02; LPPAYTNSF, HLA-B*07:02/HLA-B*08:01/HLA-C*03:03; IPTNFTISV, HLA-B*07:02/HLA-C*03:03; RAAEIRASA, HLA-B*07:02/HLA-C*03:03; TRFASVYAW, HLA-C*07:01/02; AHFPREGVF, HLA-C*07:01/02; FKNLREFVF, HLA-C*07:01/02; YQPYRVVVL, promiscuous.

Supplementary Figures

Supplementary Figure S1.

T cell IFN-γ production in response to SARS-CoV-2 peptides in unvaccinated, non-infected individuals. (A) IFNγ production (pg/mL) measured by IFNγ ELISA in conditioned media from PBMC stimulated with single SARS-CoV-2 peptides. PWUD (N=19), HD (N = 12) and Convalescent (N = 5). Each datapoint represents the response to the stimulation with one peptide. The responses to stimulation with single peptides for each donor are pooled together for each cohort. The total number of peptides tested per group is shown in the figure. Mann-Whitney U test, ****: P < 0.0001. **(B)** IFN-γ production in response to spike (N = 43) and non-spike peptides (N = 57). **(C-D)** Response breakdown by donor. Mann-Whitney U test, P > 0.05. The data shown here were obtained from the supernatants of the proliferation assay experiment shown in **Figure 1**.

Supplementary Figure S1

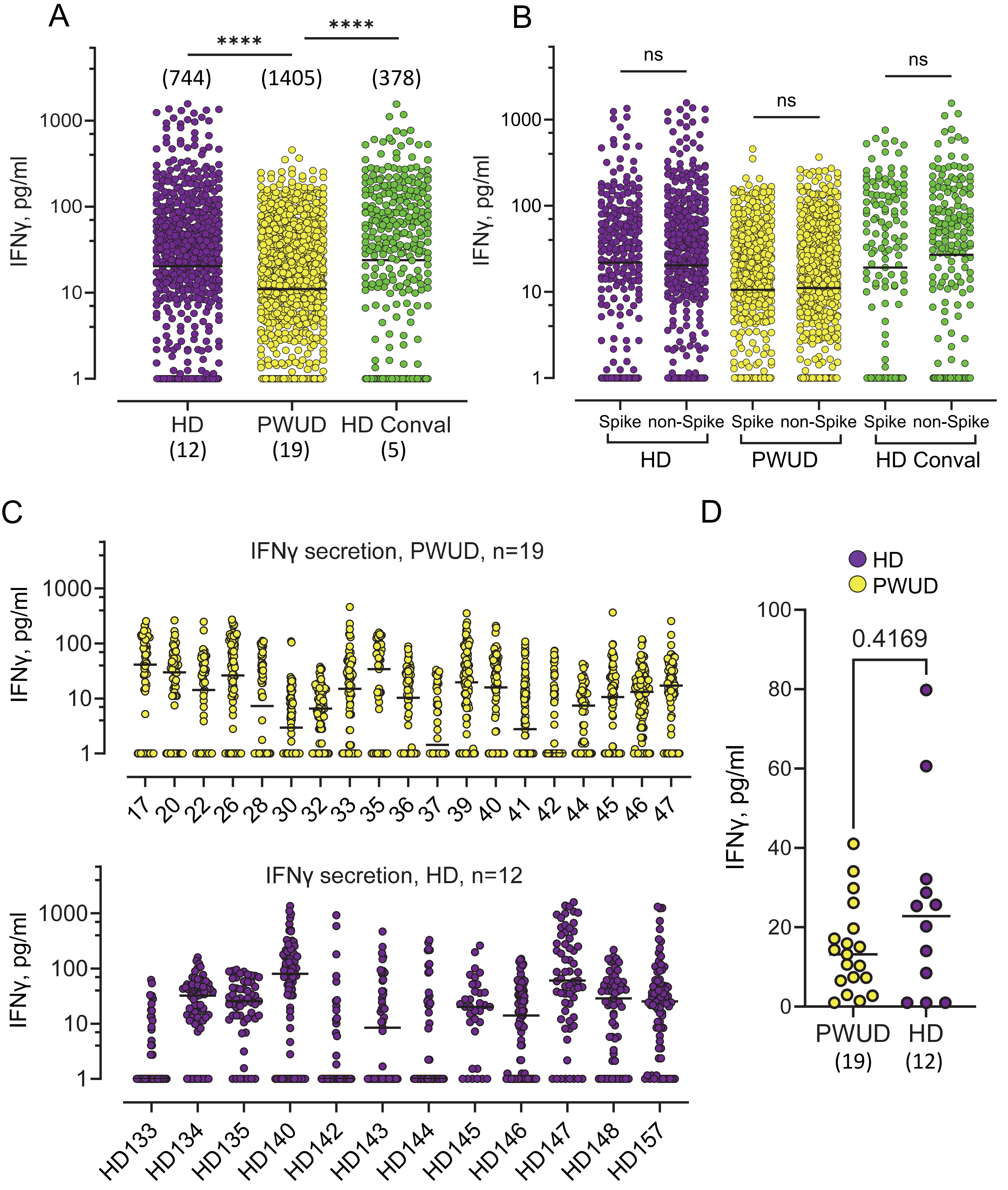

Supplementary Figure S2.

Flow cytometry gating strategy.

T cell gating is shown (FSC/SSC lymphocytes >> Singlets >> Live cells >> CD3 gate >> CD4 and CD8 gates). Examples of flow plots defined by marker combinations are shown for $CD4^+$ Th (top panels) and $CD8^+$ CTL (bottom panels) T cells.

Supplementary Figure S2

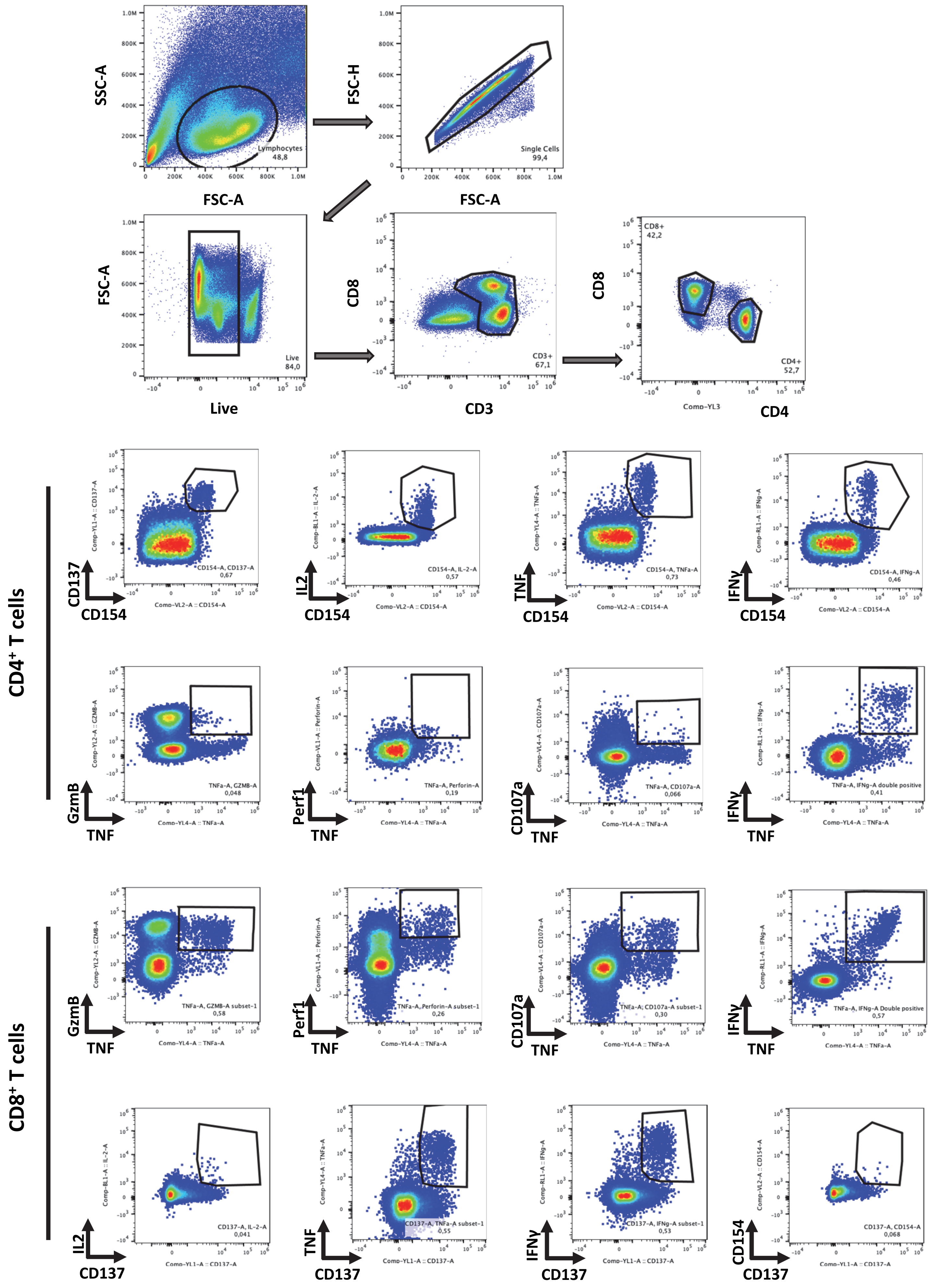

Supplementary Figure 3.

Spike-specific CD4+ T cell responses in vaccinated donors.

CD4 Th cell response to the Peptivator mix quantified by the frequency increase (over background) of cells that are double positive for $CD137^+$ $CD154^+$, $CD154^+$ $IFN\gamma^+$, $CD154^+$ $IL\text{-}2^+$, $CD154^+$ TNF^+, TNF^+ $CD107a^+$, TNF^+ $GZMB^+$, TNF^+ $IFN\gamma^+$, or TNF^+ $Perforin^+$. Mann-Whitney U test. Significant P values are shown. PWUD, N = 22; HD; N = 20. BTI = Breakthrough infection**.**

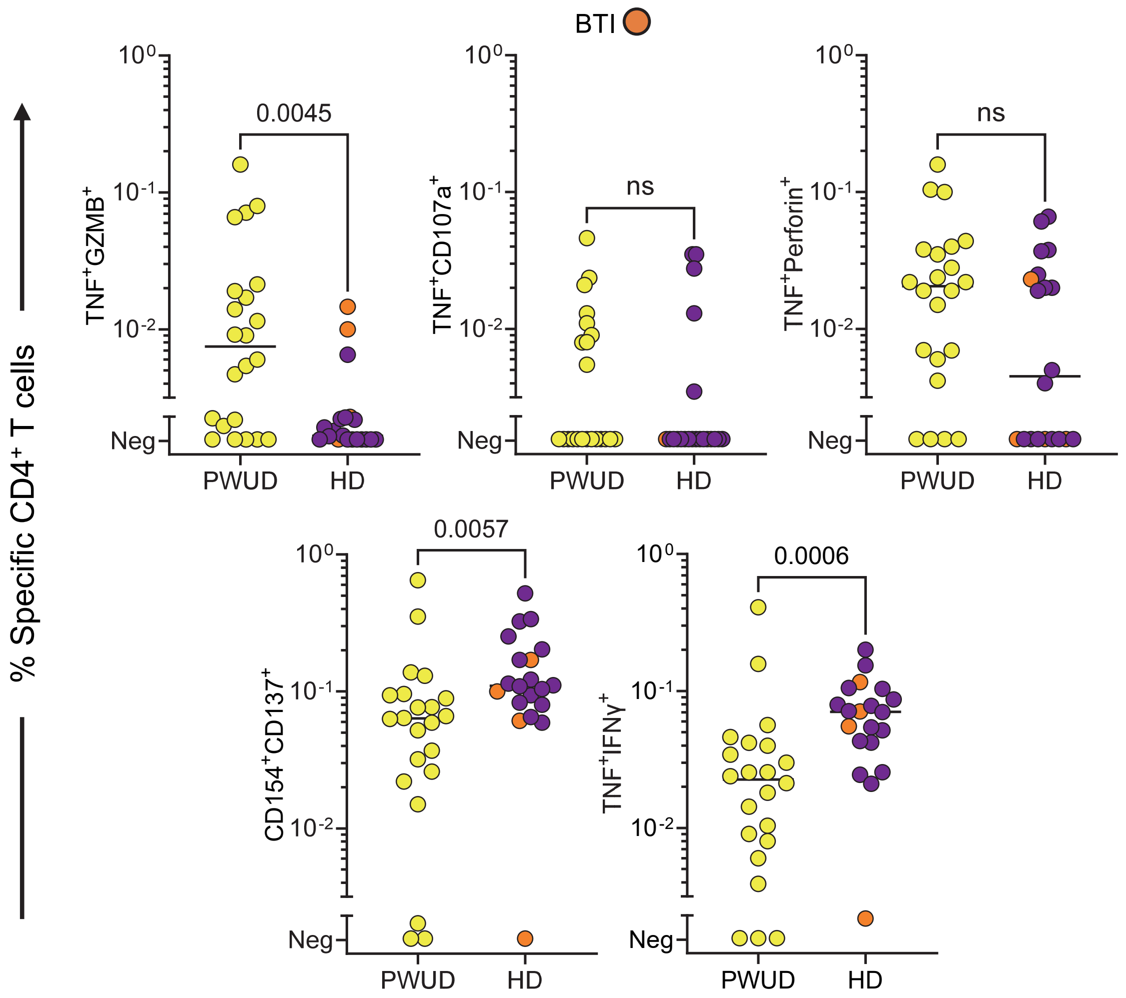

Supplementary Figure S4.

Correlation pattern of AIM combinations for $CD4^{+}$ or $CD8^{+}$ T cells.

(A-B) Pearson correlation matrix (two tailed) of the patterns of response for CD4 Th (A) and CD8 CTL cells (B) for both PWUD (left) and HD donors (right). Marker combinations and corresponding P values for the correlations are indicated.

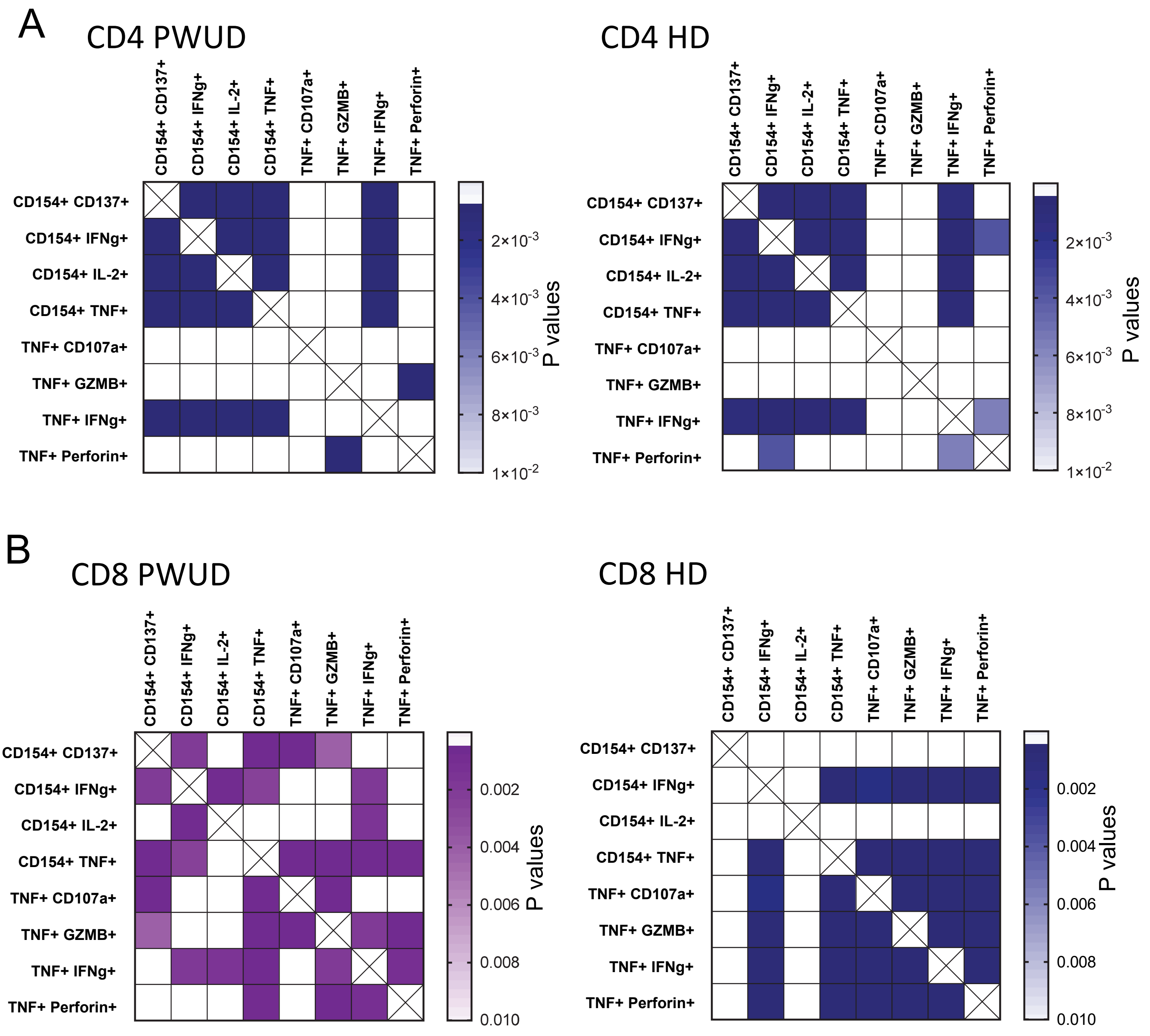

Supplementary Table S1. Patient demographics

	PWUD	HD
	N = 36	N = 33
Post-vaccination samples	25	21
Gender	M = 19, F = 6	M = 7, F = 14
Age (median)	49.5	49
Days from last dose (median)	150 days	63 days
Days from last dose (average)	149 days	123 days
mRNA vaccine Type	Pfizer, Moderna	Pfizer, Moderna
On illegal or prescribed opioids (self-reported)	17	-
Living with HIV/ on Antiretroviral Therapy	1	-
Pre-vaccination samples	19	12

Supplementary Table S2. Clinical information by patient.

PWUD donor ID	Age	Gender	On medication-assisted rehabilitation	Pre-vaccine-sampling	Post-vaccine sampling	Vaccine Type	Number of Doses	Days between vaccine doses (D1-D2)	PBMC collection (Days post vaccination)	On illegal or prescribed opioids	Living with HIV/ on antiretroviral therapy
17	47	Male	Yes (Methadone)	Yes	Yes	Pfizer	2	24	147	Yes	No
20	34	Male	Yes (Methadone)	Yes	No	-	-	-	-	Yes	No
22	48	Female	No	Yes	No	-	-	-	-	Yes	No
26	29	Female	No	Yes	Yes	Pfizer	2	55	90	Yes	No
28	35	Male	No	Yes	Yes	Pfizer	2	41	96	Yes	No
30	55	Male	No	Yes	No	-	-	-	-	Yes	No
32	49	Male	Yes (Methadone)	Yes	No	-	-	-	-	Yes	No
33	42	Male	No	Yes	Yes	Pfizer	2	30	67	Yes	No
35	60	Male	No	Yes	Yes	Pfizer	2	27	116	Did not self-report	No
36	56	Male	Yes (Methadone)	Yes	Yes	Pfizer	2	27	166	Yes	No
37	49	Female	No	Yes	No	-	-	-	-	Did not self-report	No
39	27	Male	No	Yes	Yes	Pfizer	2	24	147	Yes	No
40*	55	Male	Yes (Methadone)	Yes	Yes	Pfizer	1	-	228	Yes	No
41	57	Male	No	Yes	No	-	-	-	-	Yes	No
42	51	Male	Yes (Subutex/Buprenorphine)	Yes	No	-	-	-	-	Yes	No
44	50	Male	No	Yes	No	-	-	-	-	Yes	No
45	30	Male	No	Yes	No	-	-	-	-	Yes	No
46	29	Female	No	Yes	No	-	-	-	-	Yes	Yes
47	53	Male	Yes (Subutex/Buprenorphine)	Yes	No	-	-	-	-	Yes	No
49	36	Male	Yes (Subutex/Buprenorphine)	No	Yes	Pfizer	2	42	131	Yes	No
52	35	Male	No	No	Yes	Pfizer	2	21	171	Yes	No
59	53	Female	Yes (Buvidal/Slow release Buprenorphine)	No	Yes	Pfizer	2	48	147	Yes	No
65	27	Female	No	No	Yes	Pfizer	2	49	80	Yes	No
66	33	Male	No	No	Yes	Pfizer	2	24	168	Yes	No
68	44	Male	No	No	Yes	Pfizer	2	41	97	Yes	No
77	53	Female	No	No	Yes	Pfizer	2	21	178	Yes	No
79	56	Male	Yes (Methadone)	No	Yes	Moderna	2	42	174	Yes	No
80	59	Male	No	No	Yes	Pfizer	2	21	208	Yes	No
81	54	Male	No	No	Yes	Pfizer	2	21	208	Yes	Yes
87	52	Male	No	No	Yes	Pfizer	2	24	144	Yes	No
89	48	Male	No	No	Yes	Moderna	2	63	98	Did not self-report	No
91	55	Male	No	No	Yes	Pfizer	2	21	194	Did not self-report	No
91	53	Female	No	No	Yes	Pfizer	2	21	193	Did not self-report	No
96	45	Female	No	No	Yes	Pfizer	2	28	150	Did not self-report	No
97	51	Male	Yes (Methadone)	No	Yes	Pfizer	2	21	171	Did not self-report	No
98	54	Male	No	No	Yes	Pfizer	2	45	168	Did not self-report	No

* This person received only one dose of mRNA vaccine and was diagnosed with SARS-CoV-2 46 days before blood sampling for examination # 2

Supplementary Table S3. T cell subpopulations (SPICE categories).

	CD8 CTL cell subpopulations					CD4 Th cell subpopulations				
#	CD107	GZMB	IFN	Perforin	TNF	CD137	CD154	IFNg	IL-2	TNF
1	+	+	+	+	+	+	+	+	+	+
2	+	+	+	+	-	+	+	+	+	-
3	+	+	+	-	+	+	+	+	-	+
4	+	+	+	-	-	+	+	+	-	-
5	+	+	-	+	+	+	+	-	+	+
6	+	+	-	+	-	+	+	-	+	-
7	+	+	-	-	+	+	+	-	-	+
8	+	+	-	-	-	+	+	-	-	-
9	+	-	+	+	+	+	-	+	+	+
10	+	-	+	+	-	+	-	+	+	-
11	+	-	+	-	+	+	-	+	-	+
12	+	-	+	-	-	+	-	+	-	-
13	+	-	-	+	+	+	-	-	+	+
14	+	-	-	+	-	+	-	-	+	-
15	+	-	-	-	+	+	-	-	-	+
16	+	-	-	-	-	+	-	-	-	-
17	-	+	+	+	+	-	+	+	+	+
18	-	+	+	+	-	-	+	+	+	-
19	-	+	+	-	+	-	+	+	-	+
20	-	+	+	-	-	-	+	+	-	-
21	-	+	-	+	+	-	+	-	+	+
22	-	+	-	+	-	-	+	-	+	-
23	-	+	-	-	+	-	+	-	-	+
24	-	+	-	-	-	-	+	-	-	-
25	-	-	+	+	+	-	-	+	+	+
26	-	-	+	+	-	-	-	+	+	-
27	-	-	+	-	+	-	-	+	-	+
28	-	-	+	-	-	-	-	+	-	-
29	-	-	-	+	+	-	-	-	+	+
30	-	-	-	+	-	-	-	-	+	-
31	-	-	-	-	+	-	-	-	-	+
